# Targeted Amplicon deep sequencing of ama1 and mdr1 to track within-host *P. falciparum* diversity throughout treatment in a clinical drug trial

**DOI:** 10.12688/wellcomeopenres.17736.1

**Published:** 2022-03-16

**Authors:** Kevin Wamae, Leonard Ndwiga, Oksana Kharabora, Kelvin Kimenyi, Victor Osoti, Zaydah de Laurent, Juliana Wambua, Jennifer Musyoki, Caroline Ngetsa, Peter Kalume, Gabriel Mwambingu, Mainga Hamaluba, Rob van der Pluijm, Arjen M. Dondorp, Jeffrey Bailey, Jonathan Juliano, Philip Bejon, Lynette Ochola-Oyier

**Affiliations:** 1 Bioscience, KEMRI-Wellcome Trust Research Programme, Kilifi, Kenya; 2 Division of Infectious Diseases, Department of Medicine, School of Medicine, University of North Carolina at Chapel Hill, Chapel Hill, North Carolina, 27599, USA; 3 Centre for Tropical Medicine and Global Health, Nuffield Department of Medicine, University of Oxford, Oxford, UK; 4 Mahidol-Oxford Tropical Medicine Research Unit, Faculty of Tropical Medicine, Mahidol University, Bangkok, Thailand; 5 Department of Pathology and Laboratory Medicine, Warren Alpert Medical School, Brown University, Providence, RI, 02903, USA; 6 Department of Epidemiology, Gillings School of Global Public Health, University of North Carolina at Chapel Hill, Chapel Hill, NC, 27516, USA; 7 Curriculum in Genetics and Molecular Biology, School of Medicine, University of North Carolina at Chapel Hill, Chapel Hill, NC, USA

**Keywords:** Artemisinin-based combination therapy, pfama1, Pfmdr1, artemisinin resistance, antimalarial resistance, targeted deep sequencing, deep sequencing, msp1, msp2, glurp

## Abstract

Antimalarial therapeutic efficacy studies are routinely conducted in malaria-endemic countries to assess the effectiveness of antimalarial treatment strategies. Targeted amplicon deep sequencing (TADS) uniquely identifies and quantifies genetically distinct parasites within an infection. In this study, TADS *Plasmodium falciparum* apical membrane antigen 1 ( *ama1*), and multidrug resistance gene 1 ( *mdr1*), were used to characterize the complexity of infection (COI) and drug-resistance genotypes, respectively.

*P. falciparum* positive samples were obtained from a triple artemisinin combination therapy clinical trial conducted in 30 children under 13 years of age between 2018 and 2019 in Kilifi, Kenya. Of the 30 participants, 9 presented with recurrent parasitemia from day 26 (624h) onwards. The *ama1* and *mdr1* genes were amplified and sequenced, while *msp1, msp2 and glurp* data were obtained from the original clinical study.

The COI was comparable between *ama1* and *msp1, msp2 and glurp*, however, overall *ama1 *detected more haplotypes. Based on *ama1*, a stable number of haplotypes were detected throughout treatment up until day 3. Additionally, a recrudescent infection was identified with an *ama1* haplotype initially observed at 30h and later in an unscheduled follow-up visit. Using the relative frequencies of *ama1* haplotypes and parasitaemia, we identified a fast (<1h) and slow (>5h) clearing haplotype. As expected, only two *mdr1* haplotypes (NF and NY) were identified based on the combination of amino acid polymorphisms at codons 86 and 184.

This study highlights TADS as a sensitive tool for tracking parasite haplotypes throughout treatment and can detect variation in haplotype clearance estimates. TADS can also identify slow clearing haplotypes, a potential early sign of selection during treatment. Consequently, TADS has the capability of improving the discriminatory power to accurately distinguish recrudescences from reinfections.

## Introduction

Artemisinin-based combination therapies (ACTs) have led to high cure rates for *P. falciparum* malaria ( Bhatt *et al.*, 2015). Nonetheless, artemisinin (ART) resistance emerged and spread in Southeast (SE) Asia, evidenced as delayed parasite clearance following ACT treatment ( Ashley *et al.*, 2014; Dondorp *et al.*, 2009; Noedl *et al.*, 2008; van der Pluijm *et al.*, 2019). ART resistance facilitates the emergence of partner drug resistance since a larger number of parasites are likely to survive the three days of treatment ( van der Pluijm *et al.*, 2019). This looming threat of widespread ACT resistance would be catastrophic in sub-Saharan Africa where the burden of malaria is greatest ( WHO, 2020).

To minimize the development of drug-resistant parasites and rescue a regimen with an already failing component of ACTs, novel chemotherapeutic strategies involving the roll-out of triple artemisinin-based combination therapies (TACTs) are being evaluated ( van der Pluijm *et al.*, 2021). TACTs combine an established ACT with a second, slowly eliminated partner drug for additional antimalarial activity and protection of partner drug resistance. The potential advantage of TACTs is supported by evidence from Cambodia that artemisinin partner drugs may exert opposing selection pressures making it difficult to adapt to multiple partner drugs simultaneously ( Parobek *et al.*, 2017). The safety and efficacy of this approach have been shown in clinical trials ( Hamaluba *et al.*, 2021; van der Pluijm *et al.*, 2020) and the antimalarial therapeutic outcomes are assessed for a maximum of 42 days. Recurrent parasitaemia during this period is classified as a new (reinfection) or recrudescent infection based on molecular methods. The former is determined when genotyping methods find the recurrent parasites are distinguishable from those in the pre-treatment infection, and the latter when the parasites are indistinguishable. The standard genotyping method termed PCR correction, examines three-length polymorphic markers in parasites, namely merozoite surface protein 1 ( *msp1*), *msp2* and glutamate-rich protein ( *glurp*) ( Snounou & Beck, 1998). The amplicon sizes of these three markers are compared between pre-treatment and post-treatment parasites by either gel or capillary electrophoresis ( Liljander *et al.*, 2009; WHO, 2003). However, length polymorphic markers are met with challenges as the reliance on gel electrophoresis is limited in discriminating alleles of similar sizes (those with size differences less than 20 bp). Moreover, technical problems such as non-specific amplification ( de Valk *et al.*, 2009) and stutter peaks ( de Valk *et al.*, 2007) are some of the obstacles to using these markers to assess the number of genetically distinct parasites in an individual, also termed complexity of infection (COI). Therefore, studies that rely on these markers may underestimate parasite diversity, are insensitive to low-abundant variants and are not quantitative for relative proportions of circulating parasite clones. Targeted amplicon deep sequencing (TADS) offers high sensitivity in detecting minority parasite variants, quantifying the number of variants and their relative frequencies. TADS also offers high-throughput sequencing of *P. falciparum* diversity and drug-resistance markers ( Gruenberg *et al.*, 2019; Miller *et al.*, 2017). Apical membrane antigen 1 ( *ama1*) is a highly polyxmorphic merozoite surface antigen ( Polley & Conway, 2001) and serves as a good marker to explore parasite diversity within infections. On the other hand, mutations at codons N86Y and F184Y of the multidrug resistance gene ( *mdr1*) modulate parasite susceptibility to ACT partner drugs such as amodiaquine, lumefantrine, piperaquine and mefloquine ( Veiga *et al.*, 2016). Additionally, the rollout of ACTs has led to an increase in the *mdr1*-NFD haplotype (based on the combination of amino acid polymorphisms at codons 86, 184 and 1246) across several African studies possibly due to ACT selection pressure ( Okell *et al.*, 2018).

In this study, we examined samples from a TACT efficacy study in Kilifi ( Hamaluba *et al.*, 2021). The administration of drugs was done under observation and the patients were monitored in the hospital for the three days of treatment. Frequent blood samples were obtained for pharmacokinetic analyses that were subsequently used for TADS. The purpose of this study was to examine as a proof of concept, the utility of a genetic diversity marker ( *ama1*) combined in a single deep sequencing run with a drug resistance marker ( *mdr1*) to identify and track changes in the complexity of infections (COI) i.e., the number of *ama1* genotypes per infection as well as the *mdr1* wild-type and mutant genotypes throughout treatment.

## Methods

### Study design

*P. falciparum* positive samples were obtained from the TACT Kenya clinical trial conducted from 2018 to 2019 (ClinicalTrials.gov Identifier: NCT03452475) as described in Hamaluba *et al.* (2021). The three-drug arms were arterolane-piperaquine (ART-PQ), arterolane-piperaquine + mefloquine (ART-PQ+MF) and artemether-lumefantrine (AL). A random sample of 30 individuals was selected including all their sampling time-points: 0 hours (h), 0.5h, 1h, 2h, 3h, 4h, 6h, 8h, 12h, 18h, 24h, 30h, 36h, 42h, 48h, 72h (day 3), 168h (day 7), 336h (day 14), 504h (day 21), 672h (day 28), 840h (day 35), 1008h (day 42) and the hour of recurrent infection (REC, any sample taken during an unscheduled visit by the study participant, Table 1). 9/30 participants presented with recurrent parasitemia based on microscopy from 624h onwards making a total of 609 individual samples. This study was approved by the Oxford Tropical Research Ethics Committee in the United Kingdom and the Kenya Medical Research Institute (KEMRI) -Scientific and Ethics Review Unit (SERU).

**Table 1.** Characteristics of the study participants.

Characteristic	Antimalarial regimen			*p-value*
	ART+PQ	ART+PQ+MF	AL	
Number of Participants [n=30]	11	11	8	0.41
Median age in years [Range]	6.7	5.7	10.65	0.51
	[2.7 – 10.3]	[2.1 – 11.9]	[6.0 - 12.6]	
Gender [Females]	3	7	3	0.12
Median Parasitemia per *μ*l [Range]	142,236	78,274	90,158.50	0.7
	[8560 – 571,530]	[15,232 – 326,020]	[25,328 – 266,146]	

ART-PQ - arterolane-piperaquine, ART-PQ+MF arterolane-piperaquine + mefloquine and AL - artemether-lumefantrine.

### DNA preparation and PCR from sequencing controls and clinical samples

DNA was extracted from *P. falciparum* laboratory reference isolates, 3D7 and Dd2 (BEI Resources), and from frozen patient blood samples using the QIAamp DNA Blood Mini Kit (Qiagen) according to the manufacturer’s instructions. The DNA from 3D7 and Dd2, were mixed as follows to come up with sequencing controls: 100%:100%, 75%:25%, 85%:15%, 95%:5% and 100%:0% to determine the lowest limit of haplotype detection. Amplicons spanning *ama1* (PF3D7_1133400, nucleotides 441-946) and *mdr1* (PF3D7_0523000, nucleotides 183-719) were generated from each control and sample in duplicate using primers designed in this study (Table S1, *Extended data*) as follows: 1µl of template DNA (final amount <50ng), 0.2µl of Q5 ^®^ High-Fidelity DNA Polymerase (final concentration 0.02U/µl, New England BioLabs), 1µl (10mM) forward primers tagged with Roche ^®^ molecular identifiers (MIDs, Table S1), and reverse primers, 0.4µl of 10mM dNTPs, 4µl of 5X Q5 reaction buffer, and 12.4µl of nuclease-free water. For both *ama1* and *mdr1,* the cycling conditions were: initial denaturation (98°C - 30 sec), followed by 30 cycles of denaturation (98°C - 10 sec), annealing (60°C - 30 sec), extension (72°C - 30 sec), and final extension (72°C - 2 min). PCR products were visualised on 1% agarose gels stained with RedSafe™ Nucleic Acid Staining Solution (iNtRON Biotechnology DR). Amplicon failures were repeated with 1.5µl of template DNA.

### Amplicon Library Preparation and Sequencing

PCR amplicons were purified using the Zymo ZR-96 DNA Clean & Concentrator-5 Kit (Zymo Research) and quantified using Quant-iT™ dsDNA Assay Kit, High Sensitivity (Invitrogen), both procedures were done following the manufacturer’s instructions. Subsequently, the PCR amplicons were normalized to equal amounts of 1ng each using EB Buffer (Qiagen) and mixed to create amplicon pools of non-overlapping 26 MIDs. The KAPA Dual-Indexed Adapter Kit and the KAPA Hyper Prep Kit (Roche) were used for library preparation and the Agilent High Sensitivity D1000 ScreenTape System (5067-5584) confirmed adapter ligation. Eventually, the *ama1* and *mdr1* amplicon libraries were mixed to generate the final pool for paired-end sequencing (2x300bp chemistry) using MiSeq Reagent Kit v3 (Illumina).

### Sequence data analysis

SeekDeep v3.0.0 was used for sequence data analysis ( Hathaway *et al.*, 2018). Firstly, fastq files were demultiplexed into individual samples based on the MIDs. The paired consensus reads for each sample were trimmed and clustered to estimate the frequency of clusters (henceforth referred to as “haplotypes”). Haplotypes were discarded if they did not occur in the duplicates and if their combined relative frequency was <5%. A conservative cut-off of 5% was set based on the lowest sequencing controls’ mixture (95% 3D7 vs. 5% Dd2) unless the haplotype was independently detected in other samples at >5%. Chimeric reads were considered PCR artefacts and discarded. The relative haplotype frequency in a sample was calculated as the number of reads of each haplotype over the total number of haplotypes reads per sample. The frequency of each haplotype in the population was calculated using the total number of samples that contained the haplotype over the total number of samples genotyped. COI was defined as the number of distinct *ama1* haplotypes (varying at the nucleotide level) in each sample, while codons 86 and 184 defined the *mdr1* haplotypes. All statistical analyses were carried out in R v4.0.2 and all plots were generated using the R packages ggplot2 v3.3.1 and ggpubr v0.3.0 ( Kassambara, 2020; Wickham, 2016).

### Parasite clearance estimation

One of the early signs of slow clearing parasites is a clearance half-life that is greater than 5h ( Ashley *et al.*, 2014). Therefore, parasite clearance half-lives were calculated using the Worldwide Antimalarial Resistance Network’s (WWARN) parasite clearance estimator ( Flegg *et al.*, 2011). This was done for each of the 30 participants by extrapolating the total parasitemia to each *ama1* haplotype per infection. An estimate of the parasitaemia for each *ama1* haplotype was calculated by multiplying the parasitaemia (based on microscopy) at each time point by the frequency of each haplotype ( Mideo *et al.*, 2016) based on the number of reads per haplotype over the total number of reads per sample. These estimates were plotted as histograms.

## Results

### TADS of laboratory isolates controls

There was successful detection of the two expected *ama1* haplotypes from the 3D7 and Dd2 laboratory isolates, as well as three *mdr1* haplotypes, one from 3D7 and the 2 copies of Dd2. Additionally, both the 3D7 and Dd2 *ama1* haplotypes were detected consistently across all mixtures and in the expected proportions, including the 95%:5% mix. This provided the evidence that the assay could detect mixed infections in clinical samples, but only when the minor haplotype was at a relative frequency of ≥5% (Figure S1).

### TADS of pre-and post-treatment samples

From the 30 individuals sampled, 11 were in both the ART+PQ and ART+PQ+MF drug arms, respectively, while 8 were in the AL drug arm. There was no difference in the baseline median parasitaemia between the drug arms ( Table 1). From the available 608 samples (timepoints 0h-1008h), *ama1* and *mdr1* sequence data were successfully obtained from 330 and 233 samples, respectively (Figure S2 A and B). Samples were grouped into three categories based on parasitemia: high (>5,000), moderate (100-5,000) and low (<100 parasites per microlitre). Many samples collected between 0h-12h had high parasitemia, those collected between 18h-30h had moderate parasitemia, while those collected after 30h were primarily of low parasitemia (Figure S2C). The median read depth was 11,147 reads (range 580 –33,714) for *ama1* and 11,548 reads (range 1,022 – 55,664) for *mdr1* and consistent with decreasing parasitemia, post-treatment samples had lower sequencing success.

### Pfama1 genetic diversity during and after treatment

**Figure 1.**
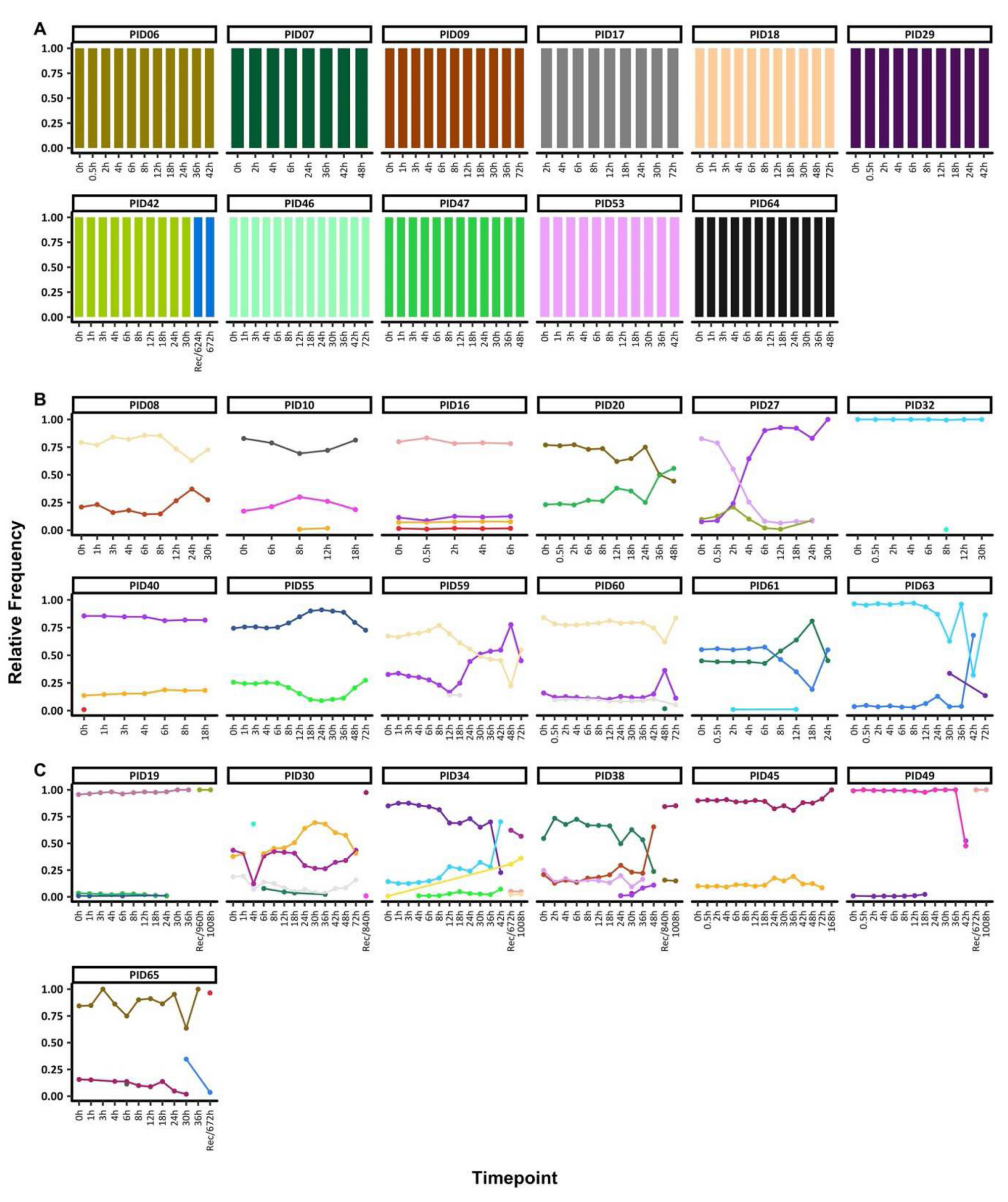
Temporal changes in *ama1* haplotypes throughout treatment. The figure highlights the individuals with **A**) monoclonal infections, if they had only one *ama1* at any timepoint **B**) polyclonal infections, if they had more than one *ama1* haplotype throughout treatment and sampled up to 72h and **C**) polyclonal infections, if they more than one *ama1* haplotype throughout treatment and sampled beyond 72h. Each coloured barplot/point/line represents a unique *ama1* haplotype and matching colours represent the same *ama1* haplotype and above each plot is the respective participant id. The x-axis represents the sampling timepoints at different hours as well as the sample at the hour of recurrence (unscheduled visit) with the prefix "Rec" while the y-axis represents the relative proportions of *ama1* haplotypes.

Throughout treatment, the mean COI (Figure S3) and number of *ama1* haplotypes ( Figure 1) were relatively stable. The mean COI and 95% confidence interval (CI) at 0h, 0.5h - 72h (during treatment) and post-treatment (after 72h) was 1.89 [1.56 – 2.22], 1.88 [1.77 – 1.99] and 1.64 [ 1.38 – 1.89], respectively. Overall, 33 *ama1* haplotypes were detected from the 330 successfully sequenced samples (Table S2) and only 10 of these haplotypes were detected at frequencies >5%.

Two groups of individuals were identified based on COI, 11 individuals with monoclonal infections compared to 19 polyclonal infections. For participants with monoclonal infections and with sequence data only up to 72h, the same *ama1* haplotype was seen throughout. There was only one exception as individual PID42 had data post-72h and there was a change in the *ama1* haplotype detected before and after 72h ( Figure 1A).

Participants with polyclonal infections harboured more than one *ama1* haplotype at any one-time point. In individuals with sequence data up to 72h (n=11), the *ama1* haplotypes detected maintained relatively stable frequencies up to <24h with most changes occurring after 24h. In five individuals (PIDs 10, 32, 40, 59 and 60), there were sporadic detections of rare *ama1* haplotypes ( Figure 1B). However, participant PID27 experienced a change in haplotype frequencies by 4h post-treatment. All the remaining 7 individuals with post-treatment data cleared their infections. However, the recurrent sample (672h) for PID65 contained an *ama1* haplotype that was present in the 30h sample, likely to be a recrudescent infection from a haplotype not detected at 0h ( Figure 1C). All participants with polyclonal infections also had polyclonal baseline samples (0h), except for PID32 who had only one polyclonal sample at 8h.

### TADS compared to msp1/msp2/glurp genotyping of recurrent infections

Based on microscopy data, nine patients had a recurrence, two in ART-PPQ, two in ART-PPQ+MF, five in AL arm and were categorized as new infections based on *msp1*/ *msp2*/ *glurp* data ( Hamaluba *et al.*, 2021). In the recurrent samples of these nine participants, *msp1/msp2/glurp* identified a total of 13, 19 and 12 haplotypes, respectively. Of these nine participants, *ama1* deep sequencing data were available for six participants and were also classified as new infections. In contrast to *msp1/msp2/glurp* genotyping, TADS identified a larger number of total haplotypes (21). For the six individuals, paired *msp1/msp2/glurp* and TADs data throughout the study revealed that the mean COI and 95% CI was highest in *msp1 =* 2.5 [0.98 – 3.5], *msp2 =* 2.5 [0.99 – 3.7], *ama1 =* 2.3 [0.81 – 3.3] and lowest in *glurp* = 1.2) during treatment (0h-72h), with the same trend post-treatment (>72h): *msp1* COI = 2.2, *msp2* (COI = 2), *ama1* (COI = 1.8) and *glurp* (COI = 1.2), and no significant difference was observed ( *p*=0.2).

### Parasite clearance estimates

**Figure 2.**
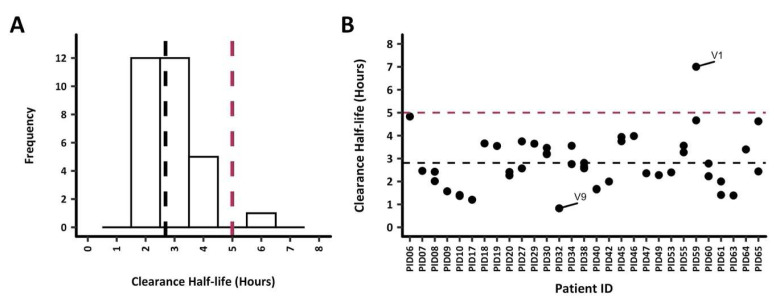
Parasite clearance estimates (PCE) for all study participants. Parasite clearance estimates for the 30 study participants are shown. The dotted lines represent the 5h clearance cut-off (red) and median clearance half-life, 2.6h (black). **A**) PCEs were calculated based on total parasitemia for each participant, the median clearance half-life was 2.7 hours. All participants had clearance half-lives <5h, however, PID59 had a clearance half-life of 5.7h. **B**) PCEs were calculated by extrapolating the clearance rates to each *ama1* haplotype (based on the parasitemia and number of reads as a basis to quantify each haplotype). The *ama1* haplotype V1 with the highest clearance half-life of 7h was from PID59. On the other hand, PID32 had the fastest clearing (0.8h) *ama1* haplotype V9.

Parasite clearance half-lives among all 30 study participants were below 5h except for PID59 who had a parasite clearance half-life of 5.7h ( Figure 2A). Nonetheless, the mean parasite clearance half-life was 2.8h for all participants. The extrapolation of the clearance rates to each *ama1* haplotype (based on the number of reads as a basis to quantify each haplotype) per infection demonstrated a similar clearance rate across most haplotypes. PID16 was excluded from any subsequent analysis since data was available for 6 hours only ( Figure 2B). Participant PID32 had one *ama1* haplotype, V9, that was cleared quite rapidly at <1h, while at least one slower clearing haplotype between 4.5h to 5h were observed in PID06 (monoclonal *ama1* infection), PID59 and PID65. PID59 also contained the slowest clearing *ama1* haplotype (V1) at 7h. There was no significant difference in the mean clearance half-lives when the haplotypes were grouped as major and minor (<5%) haplotypes (Welch two-sample t-test, *p* = 0.61).

### Pfmdr1 genetic diversity pre-and post-treatment

**Figure 3.**
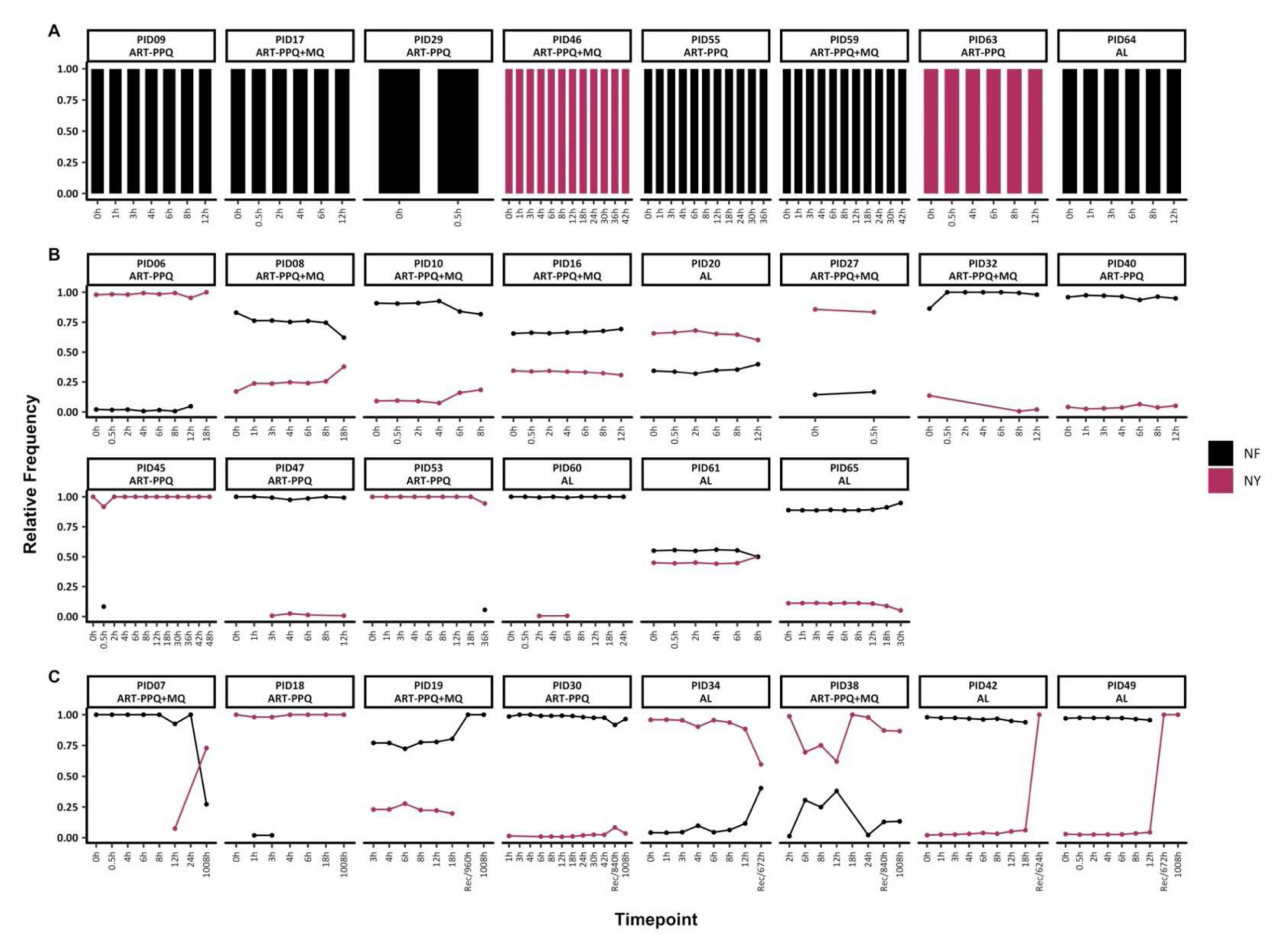
Temporal changes in *mdr1* haplotypes throughout treatment. The figure highlights the individuals with **A**) monoclonal infections, if they had only one *mdr1* at any timepoint **B**) polyclonal infections, if they had more than one *mdr1* haplotype throughout treatment and sampled up to 72h and **C**) polyclonal infections, if they more than one *mdr1* haplotype throughout treatment and sampled beyond 72h. Each coloured barplot/point/line represents a unique *mdr1* haplotype (black-NF and maroon-NY). Above each plot is the respective participant id. The x-axis represents the sampling timepoints at different hours as well as the sample at the hour of recurrence with the prefix "Rec" while the y-axis represents the relative proportions of *mdr1* haplotypes.

Based on the combination of amino acid polymorphisms at codons N86Y and F184Y, only two haplotypes (NY and NF) were detected. Of the 30 individuals with baseline data, 22 individuals harboured mixed infections with both *mdr1* haplotypes, while 8 individuals had monoclonal *mdr1* infections (either NF or NY) throughout treatment ( Figure 3A). Of the individuals with mixed *mdr1* infections, 15/22 had sequence data up to 72h only and they maintained *mdr1* haplotypes at stable frequencies, similar to *ama1*. However, in two individuals (PIDs 45 and 53) there were sporadic detections of rare *mdr1* haplotypes ( Figure 3B).

In the remaining 8/22 individuals with mixed *mdr1* infections and with post-treatment (>72h) sequence data, three of these individuals switched from a predominance of the NF haplotype to the NY haplotype during follow up either during recurrence infection (unscheduled visit) or on day 42 (1008h). Only PID18 maintained the same dominant haplotype (NY) throughout the treatment and follow-up period on day 42 (1008h) ( Figure 3C). There was no significant difference in the frequencies of Y184 and 184F haplotypes in baseline samples vs. samples collected from 12h onward (Chi-square test, *p* = 0.52).

## Discussion

Children in this moderate-high malaria transmission setting in Kilifi primarily maintained a stable homogeneous haplotype population throughout treatment until day 3 (72h). This is not surprising since febrile infections tend to occur with low COI even though with high parasite densities ( Beck *et al.*, 1997) and treatment reduces the establishment of new infections. Thus, the genetic homogeneity improves the confidence in determining reinfections post-treatment (day 28 onwards), such as the one individual identified with a haplotype at 30h and later in their recurrent infection (for an unscheduled visit at 672h). The sporadic observation of rare haplotypes at only a single time point potentially highlights the changes in parasite density, sequestration of parasites or lingering genetic material from dead parasites. The distinct changes in haplotype frequencies occurred post-treatment from day 7 onwards when re-infections are likely as the levels of drugs in the body continue to decrease. Importantly, our analysis of rare haplotypes was limited to a 5% cut-off based on sequencing controls, to increase our confidence in calling mixed infections.

The clearance rates were similar within and between individuals irrespective of clonality, suggesting that the three antimalarial treatments were equally effective in clearing haplotypes and parasitaemia. Any significant deviations in haplotype clearance rates would provide a signal of emerging resistance during treatment if a haplotype was consistently cleared at a slower rate within and between individuals. This analysis identified one such individual with an estimated slow clearance of 5.7h and a closer examination of the two main haplotypes in the infection, identified a slow clearing haplotype with an estimated clearance half-life of 7h. Thus, a drug-resistant haplotype circulating at a low frequency before treatment may survive and rapidly expand following treatment ( Ecker *et al.*, 2012; Jafari *et al.*, 2004). In addition to the slow clearing haplotype, the sole fast and three slower clearing haplotypes, indicate the variation in an individual’s ability to clear an infection and such samples need to be interrogated to determine additional genetic factors if any.

The *mdr1* genotype in codon 86 was 100% wildtype (N86). This is consistent with previous findings ( Wamae *et al.*, 2019) in the study area of a shift from 86Y to N86 by 2018 when this study began, and thus only 2 haplotypes were observed based on codon 184. Though the sample size was small, as expected, there was no selection of codon 184 with these drugs. This is in contrast to a study conducted in Tanzania between 2002 and 2004 when there was a higher frequency of *mdr1* mutant genotypes, that observed more N86 and 184F in post-treatment samples following artemether-lumefantrine treatment ( Humphreys *et al.*, 2007).

Limitations of this study include the fact the sample size across the three treatment arms was small and this may have led to biases. For example, all but one of the recurrent infections were new infections with entirely different haplotypes from the pre-treatment sample by *ama1* TADS. Therefore, it remains to be seen how sensitive TADS will be in distinguishing new vs. recurrent infections compared to *msp1*/ *mps2*/ *glurp* genotyping in larger studies. The observations made from the single individual with a slow clearing clone require further validation as this was based on data from only one individual. Moreover, since this study was set up to provide a proof of concept, it is a scalable assay that allows for additional drug resistance markers, such as k13, to be monitored. The low parasitaemia following treatment minimised the generation of good quality TADS data, impacting the sample size. Consequently, there were a limited number of samples to fully examine the changes in haplotypes throughout the study period. However, the findings were similar to several drug trials that predominantly have new rather than recrudescent infections when efficacious drugs are tested ( Adegbite *et al.*, 2019; Davlantes *et al.*, 2018; Kakolwa *et al.*, 2018). Additionally, immunity may play a role in clearing infections and thus new haplotypes are likely to be present in subsequent infections.

Improved accuracy in distinguishing infections post-treatment is important when considering the WHO recommendation of abandoning a drug if failure rates are >10% (WHO treatment guidelines), which is determined by the number of recrudescent infections that is likely to vary based on genotyping methods used and genetic markers examined. The additional analyses of tracking variants throughout treatment improve the ability to identify dominant variants pre-treatment that also appear as a new infection (in the follow-up period) and is misclassified as a recrudescent infection. The *ama1* deep sequencing yielded comparable COI to *msp1* and *msp2*, however yielding more variants than *msp1* or *msp2*. Hence, *ama1* is a highly discriminatory marker with a larger number of variants with a prevalence of <5%, providing a higher resolution to better distinguish new from recrudescent infections in moderate to high transmissions settings where polyclonal infections are common.

The assessment of genotypes throughout treatment follows the trajectory of infection, identifies the number of infecting clones and rare variants per individual. This allows for the early detection of emerging resistant variants by identifying slow clearing variants. Furthermore, the examination of drug resistance mutation frequencies during treatment can identify distinct shifts in occurrences that are likely to indicate directional selection of a rapidly rising variant ( Henriques *et al.*, 2014; Mideo *et al.*, 2013). Given the high disease burden and high levels of reinfection in sub-Saharan Africa, there is a need for improved tools for conducting molecular assays to improve the interpretation of the genotyping outputs. Such assays presented in this study, highlight a potential genotyping tool for not only PCR correction but in combination with monitoring drug resistance markers. The need for more genotyping reference labs in Africa remains to support this endeavour.

## Data availability

### Underlying data

The raw fastq files have been deposited in Zenodo under: Targeted Amplicon deep sequencing of ama1 and mdr1 to track within-host P. falciparum diversity in Kilifi, KENYA (Version 1) [Data set]. Zenodo. DOI: https://doi.org/10.5281/zenodo.6243929 ( Wamae *et al.*, 2022a)

The nucleotide sequence data reported in this paper are available in the GenBank database under the accession numbers: *ama1* (MZ593448 - MZ593480) and *mdr1* (MZ593481 - MZ593484).

Data are available under the terms of the Creative Commons Attribution 4.0 International license (CC-BY 4.0).

### Extended data

Extended data tables and figures have been deposited in Zenodo under: Targeted Amplicon deep sequencing of ama1 and mdr1 to track within-host P. falciparum diversity throughout treatment in a clinical drug trial (Version 1) [Data set]. DOI: https://doi.org/10.5281/zenodo.6253570 ( Wamae *et al.*, 2022b).

This collection contains the following extended data:

- **Table S1. List of PCR and deep sequencing primers**. This table shows the list of forward and reverse primers used for deep sequencing. In boldface are the MID tags while in the regular face are the forward primers.
- **Table S2. The relative frequencies of each ama1 variant and the number of samples with each variant** *.* The relative frequencies (%) of the 33 AMA1 variants in pre-and post-treatment samples (n = 330) are shown as a 33 amino acid sequence. The frequencies were calculated by dividing the number of reads of each haplotype by the total number of reads obtained (116,187,131).
- **Figure S1. Performance of TADS on the sequencing controls made up of a mixture of laboratory isolates.** Each control set was done in 6 replicates to ensure sufficient control data in the event of potential amplicon or sequencing failure. The median read depth in the lab controls was 5658 (range 4,310 – 12,603) and 658 (- 1,676). The x-axis represents the replicate identifier across the five mixtures, starting from 1 to 6 while the y-axis represents the proportions of each variant across all replicates. For ama1 (A), 2 variants (3D7 and Dd2) were detected whereas in MDR1 (B), 3 variants were detected YY, FY and NY following amplification of Dd2 Copy I, Dd2 Copy II and 3D7, respectively. For *ama1*, sequencing failed for replicate 6 of control set 1, while for *mdr1*, sequencing failed for replicate 2 and 6 of control set 3, replicates 1 and 6 of control set 4 and replicates 1 and 5 of control set 5. Under the MDR1 control set 4, the Dd2 copy II (86F, 184Y) was not identified possibly due to having very low concentrations that were not picked up in this replicate. Based on our control mixtures, the minimum variant frequency we were able to detect was 0.5%.
- **Figure S2. Heatmaps of the successfully PCR amplified and sequenced samples for *ama1* (A) and *mdr1* (B).** The rows represent the study participants while the columns represent time in hours. Successfully sequenced samples are shown in green, those that failed PCR in red and those that failed sequencing are in yellow. The timepoint Rec, represent unscheduled visits where a recurrent sample was collected. The unshaded areas with ‘-‘ are timepoints where samples were not collected. For each time-point, the number of samples successfully sequenced, n, is indicated in the last row of each panel. The table in panel C shows the groupings of samples based on parasitemia, high (> 5,000), moderate (100-5,000) and low (< 100 parasites per microlitre). Many samples collected between 0h-12h had high parasitemia, samples collected between 18h-30h had moderate parasitemia while samples collected after 30h were primarily of low parasitemia.
- **Figure S3. The mean complexity of infection (COI) by AMA1 throughout treatment.** The mean COI (red diamonds) appeared to be stable (between 1.5 - 2) from baseline (0h) up to 72h and thereafter fluctuates due to the small sample sizes (<5) in the post-treatment samples. The black dots represent the COI per sample.

Data are available under the terms of the Creative Commons Attribution 4.0 International license (CC-BY 4.0).
