# Targeted amplicon deep sequencing of ama1 and mdr1 to track within-host *P. falciparum* diversity throughout treatment in a clinical drug trial

**DOI:** 10.12688/wellcomeopenres.17736.2

**Published:** 2022-12-15

**Authors:** Kevin Wamae, Leonard Ndwiga, Oksana Kharabora, Kelvin Kimenyi, Victor Osoti, Zaydah de Laurent, Juliana Wambua, Jennifer Musyoki, Caroline Ngetsa, Peter Kalume, Gabriel Mwambingu, Mainga Hamaluba, Rob van der Pluijm, Arjen M. Dondorp, Jeffrey Bailey, Jonathan Juliano, Philip Bejon, Lynette Ochola-Oyier

**Affiliations:** 1 Bioscience, KEMRI-Wellcome Trust Research Programme, Kilifi, Kenya; 2 Division of Infectious Diseases, Department of Medicine, School of Medicine, University of North Carolina at Chapel Hill, Chapel Hill, North Carolina, 27599, USA; 3 Centre for Tropical Medicine and Global Health, Nuffield Department of Medicine, University of Oxford, Oxford, UK; 4 Mahidol-Oxford Tropical Medicine Research Unit, Faculty of Tropical Medicine, Mahidol University, Bangkok, Thailand; 5 Department of Pathology and Laboratory Medicine, Warren Alpert Medical School, Brown University, Providence, RI, 02903, USA; 6 Department of Epidemiology, Gillings School of Global Public Health, University of North Carolina at Chapel Hill, Chapel Hill, NC, 27516, USA; 7 Curriculum in Genetics and Molecular Biology, School of Medicine, University of North Carolina at Chapel Hill, Chapel Hill, NC, USA

**Keywords:** Artemisinin-based combination therapy, pfama1, Pfmdr1, artemisinin resistance, antimalarial resistance, targeted deep sequencing, deep sequencing, msp1, msp2, glurp

## Abstract

**Introduction:** Antimalarial therapeutic efficacy studies are routinely conducted in malaria-endemic countries to assess the effectiveness of antimalarial treatment strategies. Targeted amplicon sequencing (AmpSeq) uniquely identifies and quantifies genetically distinct parasites within an infection. In this study, AmpSeq of *Plasmodium falciparum* apical membrane antigen 1 ( *ama1*), and multidrug resistance gene 1 ( *mdr1*), were used to characterise the complexity of infection (COI) and drug-resistance genotypes, respectively.

**Methods:** *P. falciparum*-positive samples were obtained from a triple artemisinin combination therapy clinical trial conducted in 30 children under 13 years of age between 2018 and 2019 in Kilifi, Kenya. Nine of the 30 participants presented with recurrent parasitemia from day 26 (624h) onwards. The *ama1* and *mdr1* genes were amplified and sequenced, while *msp1, msp2 and glurp* data were obtained from the original clinical study.

**Results:** The COI was comparable between *ama1* and *msp1, msp2 and glurp*; overall, *ama1 *detected more microhaplotypes. Based on *ama1*, a stable number of microhaplotypes were detected throughout treatment until day 3. Additionally, a recrudescent infection was identified with an *ama1* microhaplotype initially observed at 30h and later in an unscheduled follow-up visit. Using the relative frequencies of *ama1* microhaplotypes and parasitemia, we identified a fast (<1h) and slow (>5h) clearing microhaplotype. As expected, only two *mdr1* microhaplotypes (NF and NY) were identified based on the combination of amino acid polymorphisms at codons 86 and 184.

**Conclusions:** This study highlights AmpSeq as a tool for highly-resolution tracking of parasite microhaplotypes throughout treatment and can detect variation in microhaplotype clearance estimates. AmpSeq can also identify slow-clearing microhaplotypes, a potential early sign of selection during treatment. Consequently, AmpSeq has the capability of improving the discriminatory power to distinguish recrudescences from reinfections accurately.

## Introduction

Artemisinin-based combination therapies (ACTs) have led to high cure rates for *P. falciparum* malaria ( Bhatt *et al.*, 2015). Still, artemisinin (ART) resistance emerged and spread in Southeast (SE) Asia, evidenced by delayed parasite clearance following ACT treatment ( Ashley *et al.*, 2014; Dondorp *et al.*, 2009; Noedl *et al.*, 2008; van der Pluijm *et al.*, 2019). Two recent studies have identified early signs of ART partial resistance in Rwanda ( Uwimana *et al.*, 2021) and Uganda ( Balikagala *et al.*, 2021), and this looming threat of widespread ACT resistance would be catastrophic in sub-Saharan Africa, where the burden of malaria is the most significant ( WHO, 2021a). 

To minimise the development of drug-resistant parasites and rescue a regimen with an already failing component of ACTs, novel chemotherapeutic strategies involving the roll-out of triple artemisinin-based combination therapies (TACTs) are being evaluated ( van der Pluijm *et al.*, 2021). TACTs combine an established ACT with a second, slowly eliminated partner drug for additional antimalarial activity and protection of partner drug resistance. The potential advantage of TACTs is supported by evidence from Cambodia that artemisinin partner drugs may exert opposing selection pressures making it difficult to adapt to multiple partner drugs simultaneously ( Parobek *et al.*, 2017). The safety and efficacy of this approach have been shown in clinical trials ( Hamaluba *et al.*, 2021; van der Pluijm *et al.*, 2020), and the antimalarial therapeutic outcomes are assessed for a maximum of 42 days. Based on molecular methods, recurrent parasitemia during this period is classified as a new (reinfection) or recrudescent infection. The former is determined when genotyping methods find the recurrent parasites are distinguishable from those in the pre-treatment infection, and the latter when the parasites are indistinguishable. The standard genotyping method termed PCR correction examines three-length polymorphic markers in parasites, namely merozoite surface protein 1 ( *msp1*), *msp2* and glutamate-rich protein ( *glurp*) ( Snounou & Beck, 1998). The amplicon sizes of these three markers are compared between pre-treatment and post-treatment parasites by either gel or capillary electrophoresis ( Liljander *et al.*, 2009; WHO, 2003). However, the use of *msp1*/ *msp2*/ *glurp* genotyping is met with challenges such as the reliance on gel electrophoresis being limited to discriminating alleles of similar sizes (those with size differences less than 20 bp) and the inability to detect low-density parasite clones ( Felger *et al.*, 2020; Jones *et al.*, 2021). Therefore, studies that rely on these markers may underestimate parasite diversity, are insensitive to low-abundant variants and are not quantitative for relative proportions of circulating parasite clones. Targeted amplicon sequencing, referred to as amplicon sequencing (AmpSeq) from here henceforth, offers high sensitivity in detecting minority parasite variants, quantifying the number of variants and their relative frequencies. AmpSeq also offers high-throughput sequencing of *P. falciparum* diversity and drug-resistance markers ( Gruenberg *et al.*, 2019; Miller *et al.*, 2017). Apical membrane antigen 1 ( *ama1*) is a highly polymorphic merozoite surface antigen ( Polley & Conway, 2001) and serves as an excellent marker to explore parasite diversity within infections. On the other hand, mutations at codons N86Y and F184Y of the multidrug resistance gene ( *mdr1*) modulate parasite susceptibility to ACT partner drugs such as amodiaquine, lumefantrine, piperaquine and mefloquine ( Veiga *et al.*, 2016). Additionally, the rollout of ACTs has led to an increase in the *mdr1*-NFD microhaplotype (based on the combination of amino acid polymorphisms at codons 86, 184 and 1246) across several African studies, possibly due to ACT selection pressure ( Okell *et al.*, 2018).

In this study, we examined samples from a TACT efficacy study in Kilifi ( Hamaluba *et al.*, 2021). The administration of drugs was done under observation, and the patients were monitored in the hospital for three days of treatment. Frequent blood samples were obtained for pharmacokinetic analyses that were subsequently used for AmpSeq. The purpose of this study was to examine the utility of a genetic diversity marker ( *ama1*) combined in a single deep sequencing run with a drug resistance marker ( *mdr1*) to identify and track changes in the complexity of infections (COI), i.e., the number of *ama1* genotypes per infection as well as the *mdr1* wild-type and mutant genotypes throughout treatment.

## Methods

### Study design

*P. falciparum* positive samples were obtained from the TACT Kenya clinical trial conducted from 2018 to 2019 (ClinicalTrials.gov Identifier: NCT03452475) described in Hamaluba *et al.* (2021). The three-drug arms were arterolane-piperaquine (ART-PQ), arterolane-piperaquine + mefloquine (ART-PQ+MF) and artemether-lumefantrine (AL). A random sample of 30 individuals was selected, including all their sampling time-points: 0 hours (h), 0.5h, 1h, 2h, 3h, 4h, 6h, 8h, 12h, 18h, 24h, 30h, 36h, 42h, 48h, 72h (day 3), 168h (day 7), 336h (day 14), 504h (day 21), 672h (day 28), 840h (day 35), 1008h (day 42) and the hour of recurrent infection (REC, any sample taken during an unscheduled visit by the study participant, Table 1). 9/30 participants presented with recurrent parasitemia based on microscopy from 624h onwards, making a total of 609 individual samples. This study was approved by the Oxford Tropical Research Ethics Committee in the United Kingdom and the Kenya Medical Research Institute (KEMRI) -Scientific and Ethics Review Unit (SERU).

**Table 1.** Characteristics of the study participants.

Characteristic	Antimalarial regimen			*p-value*
	ART+PQ	ART+PQ+MF	AL	
Number of Participants [n=30]	11	11	8	0.41
Median age in years [Range]	6.7	5.7	10.65	0.51
	[2.7 – 10.3]	[2.1 – 11.9]	[6.0 - 12.6]	
Gender [Females]	3	7	3	0.12
Median Parasitemia per *μ*l [Range]	142,236	78,274	90,158.5	0.7
	[8560 – 571,530]	[15,232 – 326,020]	[25,328 – 266,146]	

ART-PQ - arterolane-piperaquine, ART-PQ+MF arterolane-piperaquine + mefloquine and AL - artemether-lumefantrine.

### DNA preparation and PCR from sequencing controls and clinical samples

Henceforth, we use the term “microhaplotype” to refer to the set of amino acid polymorphisms found on a single DNA amplicon. DNA was extracted from culture-adapted laboratory *P. falciparum* isolates, 3D7 and Dd2 (BEI Resources), and from 200μl of frozen patient blood samples using the QIAamp DNA Blood Mini Kit (Qiagen) according to the manufacturer’s instructions. The DNA from 3D7 and Dd2 were mixed in the following ratios to generate artificial mixtures of sequencing controls: 0.5:0.5, 0.75:0.25, 0.85:0.15, 0.95:0.05 and 1:0 to determine the lowest limit of a microhaplotype detection. The sequencing controls allowed for the detection of two ama1 (3D7 and Dd2) and three mdr1 (one from 3D7 and two from Dd2) microhaplotypes. Dd2 contained two mdr1 gene copies generated due to adaptation to in vitro culture. Amplicons spanning *ama1* (PF3D7_1133400, nucleotides 441–946) and *mdr1* (PF3D7_0523000, nucleotides 183–719) were generated in duplicate from each control and sample, using primers designed in this study (Table S1) as follows: 1µl of template DNA (final amount <50ng), 0.2µl of Q5 ^®^ High-Fidelity DNA Polymerase (final concentration 0.02U/µl, New England BioLabs), 1µl (10mM) forward primers tagged with Roche ^®^ molecular identifiers (MIDs, Table S1), and reverse primers, 0.4µl of 10mM dNTPs, 4µl of 5X Q5 reaction buffer, and 12.4µl of nuclease-free water. For both *ama1* and *mdr1,* the cycling conditions were: initial denaturation (98°C - 30 sec), followed by 30 cycles of denaturation (98°C - 10 sec), annealing (60°C - 30 sec), extension (72°C - 30 sec), and final extension (72°C - 2 min). PCR products were visualised on 1% agarose gels stained with RedSafe™ Nucleic Acid Staining Solution (iNtRON Biotechnology DR). Amplicon failures were repeated with 1.5µl of template DNA.

### Amplicon Library Preparation and Sequencing

PCR amplicons were purified using the Zymo ZR-96 DNA Clean & Concentrator-5 Kit (Zymo Research) and quantified using Quant-iT™ dsDNA Assay Kit, High Sensitivity (Invitrogen). Both procedures were done following the manufacturer’s instructions. Subsequently, the PCR amplicons were normalised to equal amounts of 1ng each using EB Buffer (Qiagen) and mixed to create amplicon pools of non-overlapping 26 MIDs. The KAPA Dual-Indexed Adapter Kit and the KAPA Hyper Prep Kit (Roche) were used for library preparation, and the Agilent High Sensitivity D1000 ScreenTape System (5067-5584) confirmed adapter ligation. Eventually, the *ama1* and *mdr1* amplicon libraries were mixed to generate the final pool for paired-end sequencing (2x300bp chemistry) using MiSeq Reagent Kit v3 (Illumina).

### Sequence data analysis

Data extraction, quality control processing, and microhaplotype clustering were performed using SeekDeep v3.0.0 ( Hathaway *et al.*, 2018). Microhaplotypes were discarded if they did not occur in the two PCR replicates and if their combined relative frequency was <5%. A conservative cut-off of 5% was set based on the isolate with the least proportion in one of the artificial mixtures of sequencing controls (ratio of 0.95 3D7 to 0.05 Dd2) unless the microhaplotype was independently detected in other samples at >5%. Chimeric reads were considered PCR artefacts and discarded. The relative microhaplotype frequency in a sample was calculated as the number of reads of each microhaplotype over the total number of reads per sample. COI was defined as the number of distinct *ama1* microhaplotypes (varying at the nucleotide level) in each sample, while codons 86 and 184 defined the *mdr1* microhaplotypes. *ama1* expected heterozygosity ( *H _e_*) was calculated using the formula below, where n is the sample size and *p _i_* is the relative frequency of the i ^th^ microhaplotype in the population ( Nei, 1978):


$$h=\frac{n}{n-1}(1-\sum p_{i}^{2})$$

*msp1/msp2/glurp* genotyping was performed according to the WHO-recommended method of gel electrophoresis ( WHO, 2008). These data were obtained from the original study, and the following was ensured during the analysis: Each PCR product had to have well-defined and easy to visualise, bands had to be bright and sharp to be of sufficient quality for scoring, PCRs were repeated if bands appeared in the negative control, the interpretation of results did not include products with less than 100 bp and did not account for faint bands or bands that formed smile-shaped patterns on the gel ( Hamaluba *et al.*, 2021). All statistical analyses were carried out in R v4.0.2, and all plots were generated using the R packages ggplot2 v3.3.1 and ggpubr v0.3.0 ( Kassambara, 2020; Wickham, 2016).

Based on simulation studies of amplicon sequencing data analysis ( Jones *et al.*, 2021), we set the lower sampling limit for a parasite (blood sampling limit) at ten parasites/μl. Finally, using the complexity of infection for each sample, we back-calculated each parasite isolate’s parasitemia to determine which isolates were at risk of falling below the sampling limit.

### Parasite clearance estimation

One of the early signs of slow clearing parasites is a clearance half-life greater than five hours ( Ashley *et al.*, 2014). Therefore, parasite clearance half-lives were calculated using the Worldwide Antimalarial Resistance Network’s (WWARN) parasite clearance estimator ( Flegg *et al.*, 2011). This was done for the 30 participants by extrapolating the total parasitemia to each *ama1* microhaplotype per infection. An estimate of the parasitaemia for each *ama1* microhaplotype was calculated by multiplying the parasitaemia (based on microscopy) at each time point by the frequency of each microhaplotype ( Mideo *et al.*, 2016) based on the number of reads per microhaplotype over the total number of reads per sample. These estimates were plotted as histograms.

## Results

### AmpSeq in artificial mixtures of sequencing controls

The expected microhaplotypes were successfully detected from the sequencing controls (two from *ama1* and three from *mdr1*). Additionally, the 3D7 and Dd2 *ama1* microhaplotypes were detected consistently across all mixtures and in the expected proportions. This provided evidence that the assay could detect mixed infections in clinical samples, but only when the minor microhaplotype was at a relative frequency of ≥5% (Figure S1).

### AmpSeq of pre-and post-treatment samples

From the 30 individuals sampled, 11 were in both the ART+PQ and ART+PQ+MF drug arms, respectively, while 8 were in the AL drug arm. There was no difference in the baseline median parasitaemia between the drug arms ( Table 1). From the available 608 samples (timepoints 0h–1008h), *ama1* and *mdr1* sequence data were successfully obtained from 330 and 233 samples, respectively (Figure S2A and B). Samples were grouped into three categories based on parasitemia: high (>5,000), moderate (100–5,000) and low (<100 parasites/μl). Many samples collected between 0h–12h had high parasitemia, those collected between 18h–30h had moderate parasitemia, while those collected after 30h were primarily low parasitemia (Figure S2C). The median read depth was 11,147 reads (range 580 –33,714) for *ama1* and 11,548 reads (range 1,022 – 55,664) for *mdr1*, consistent with decreasing parasitemia, post-treatment samples had lower sequencing success.

### *Pfama1* genetic diversity during and after treatment

**Figure 1.**
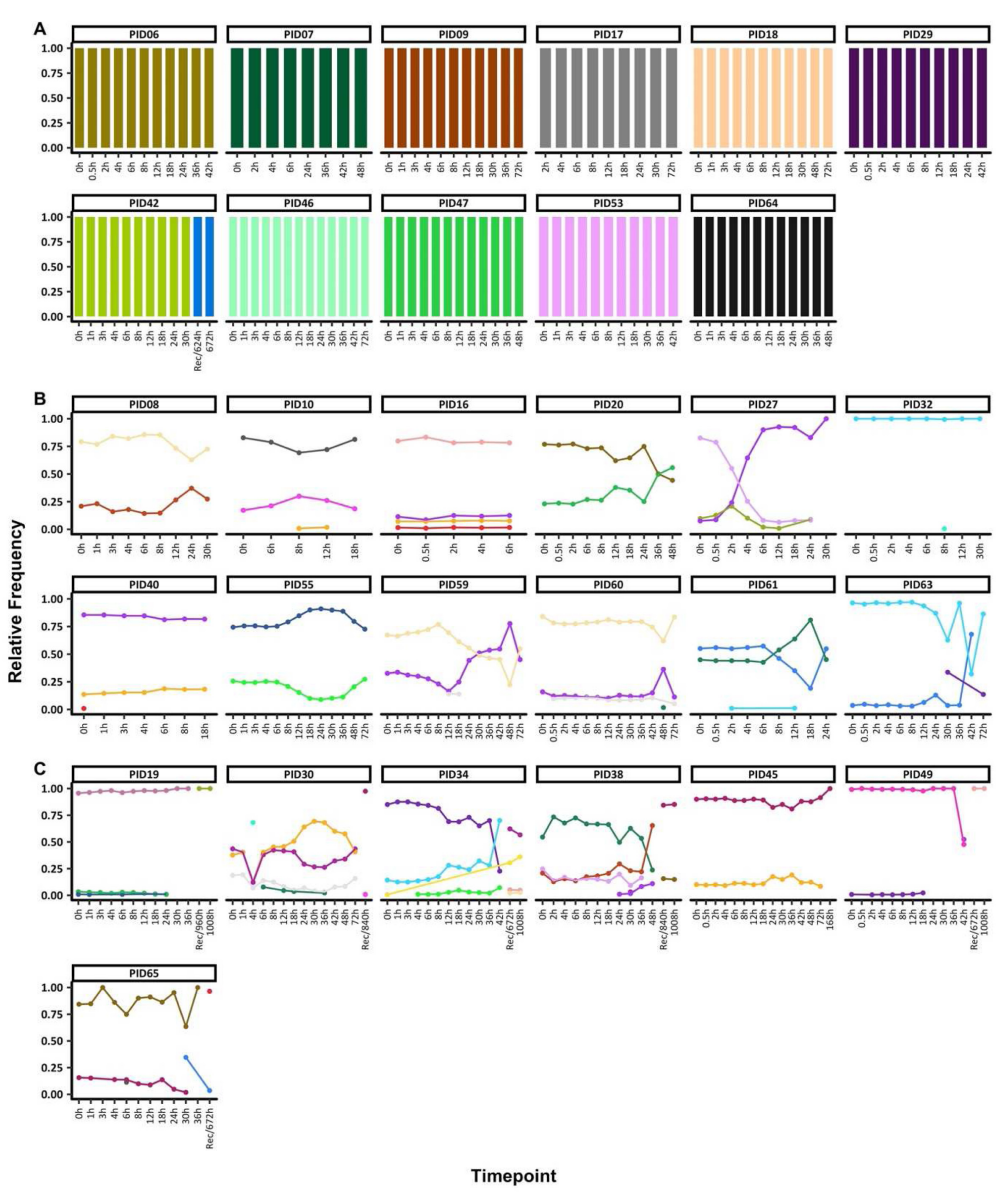
Temporal changes in *ama1* microhaplotypes throughout treatment. The figure highlights the individuals with **A**) monoclonal infections, if they had only one *ama1* at any timepoint **B**) polyclonal infections, if they had more than one *ama1* microhaplotype throughout treatment and sampled up to 72h and **C**) polyclonal infections, if they more than one *ama1* microhaplotype throughout treatment and sampled beyond 72h. Each coloured barplot/point/line represents a unique *ama1* microhaplotype and matching colours represent the same *ama1* microhaplotype and above each plot is the respective participant id. The x-axis represents the sampling timepoints at different hours as well as the sample at the hour of recurrence (unscheduled visit) with the prefix "Rec" while the y-axis represents the relative proportions of *ama1* microhaplotypes.

Throughout treatment, the mean COI (Figure S3) and number of *ama1* microhaplotypes ( Figure 1) were relatively stable and the expected heterozygosity for *ama1* was high, 0.96. The mean COI and 95% confidence interval (CI) at 0h, 0.5h – 72h (during treatment) and post-treatment (after 72h) was 2.44 [2.13-1.75], 2.37 [2.27-2.48] and 2.0 [1.28-2.7], respectively. Overall, 33 *ama1* microhaplotypes were detected from the 330 successfully sequenced samples (Table S2), and only 10 of these microhaplotypes were detected at frequencies >5%.

Two groups of individuals were identified based on COI, 11 individuals with monoclonal infections compared to 19 polyclonal infections. For participants with monoclonal infections and with sequence data only up to 72h, the same *ama1* microhaplotype was seen throughout. Only one exception, individual PID42 had data post-72h and a change in the *ama1* microhaplotype was detected before and after 72h ( Figure 1A).

Participants with polyclonal infections harboured more than one *ama1* microhaplotype at any one-time point. In individuals with sequence data up to 72h (n=11), the *ama1* microhaplotypes detected maintained relatively stable frequencies up to <24h, with most changes occurring after 24h. In five individuals (PIDs 10, 30, 32, 40, 59, 60 and 61), there were sporadic detections of rare *ama1* microhaplotypes ( Figure 1B), including one microhaplotype each in PID10 at 8h and 12h, PID 30 at 4h, PID32 at 8h, PID40 at 0h, PID59 at 12h and 18h, PID60 at 48h, PID61 at 12h and PID65 at 6h. Except for PID60, all these sporadic microhaplotypes were detected above the sampling limit of 10 parasites/μl (Table S3).

Participant PID27 experienced a drastic change in the relative microhaplotype frequencies by 4h post-treatment. The least dominant microhaplotype at 0h had become dominant by the 4h and remained this way up to the last timepoint with sequencing data (30h). All the remaining seven individuals with post-treatment data cleared their infections. However, the recurrent sample (672h) for PID65 contained an *ama1* microhaplotype present in the 30h sample, likely from a minor microhaplotype not detected at 0h ( Figure 1C). All participants with polyclonal infections also had polyclonal baseline samples (0h), except for PID32 who had only one polyclonal sample at 8h.

### AmpSeq compared to msp1/msp2/glurp genotyping of recurrent infections

Based on microscopy data, all the nine individuals recurrences (two in ART-PPQ, two in ART-PPQ+MF, and five in the AL arm) were categorised as new infections based on *msp1*/ *msp2*/ *glurp* data ( Hamaluba *et al.*, 2021). *msp1/msp2/glurp* identified a total of 13, 19 and 12 microhaplotypes, respectively, in the recurrent samples of these nine participants. Of these nine participants, *ama1* deep sequencing data were available for six participants and were also classified as new infections. In contrast to *msp1/msp2/glurp* genotyping, AmpSeq identified 21 *ama1* microhaplotypes, slightly more than the other markers. For the six individuals, paired *msp1/msp2/glurp* and AmpSeq data revealed that the mean COI and 95% CI was highest in *msp1 =* 2.5 [0.98 – 3.5], *msp2 =* 2.5 [0.99 – 3.7], *ama1 =* 2.3 [0.81 – 3.3] and lowest in *glurp* = 1.2) during treatment (0h-72h), with the same trend post-treatment (>72h): *msp1* COI = 2.2, *msp2* (COI = 2), *ama1* (COI = 1.8) and *glurp* (COI = 1.2), and no significant difference was observed ( *p*=0.2).

### Parasite clearance estimates

**Figure 2.**
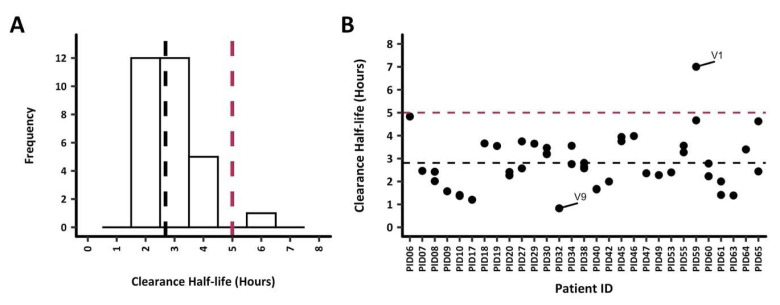
Parasite clearance estimates (PCE) for all study participants. Parasite clearance estimates for the 30 study participants are shown. The dotted lines represent the 5h clearance cut-off (red) and median clearance half-life, 2.6h (black). **A**) PCEs were calculated based on total parasitemia for each participant, the median clearance half-life was 2.7 hours. All participants had clearance half-lives <5h, however, PID59 had a clearance half-life of 5.7h. **B**) PCEs were calculated by extrapolating the clearance rates to each *ama1* microhaplotype (based on the parasitemia and number of reads to quantify each microhaplotype). The *ama1* microhaplotype V1 with the highest clearance half-life of 7h was from PID59. On the other hand, PID32 had the fastest clearing (0.8h) *ama1* microhaplotype V9.

Parasite clearance half-lives among all 30 study participants were below 5h except for PID59, with a parasite clearance half-life of 5.7h ( Figure 2A). Nonetheless, the mean parasite clearance half-life was 2.8h for all participants. The extrapolation of the clearance rates to each *ama1* microhaplotype (based on the number of reads to quantify each microhaplotype) per infection demonstrated a similar clearance rate across most microhaplotypes. PID16 was excluded from any subsequent analysis since data was available for 6 hours only ( Figure 2B). Participant PID32 had one *ama1* microhaplotype, V9, that was cleared quite rapidly at <1h, while at least one slower clearing microhaplotype between 4.5h to 5h was observed in PID06 (monoclonal *ama1* infection), PID59 and PID65. PID59 also contained the slowest clearing *ama1* microhaplotype (V1) at 7h. There was no significant difference in the mean clearance half-lives when the microhaplotypes were grouped as major and minor (<5%) microhaplotypes (Welch two-sample t-test, *p* = 0.61).

### *Pfmdr1* genetic diversity pre-and post-treatment

**Figure 3.**
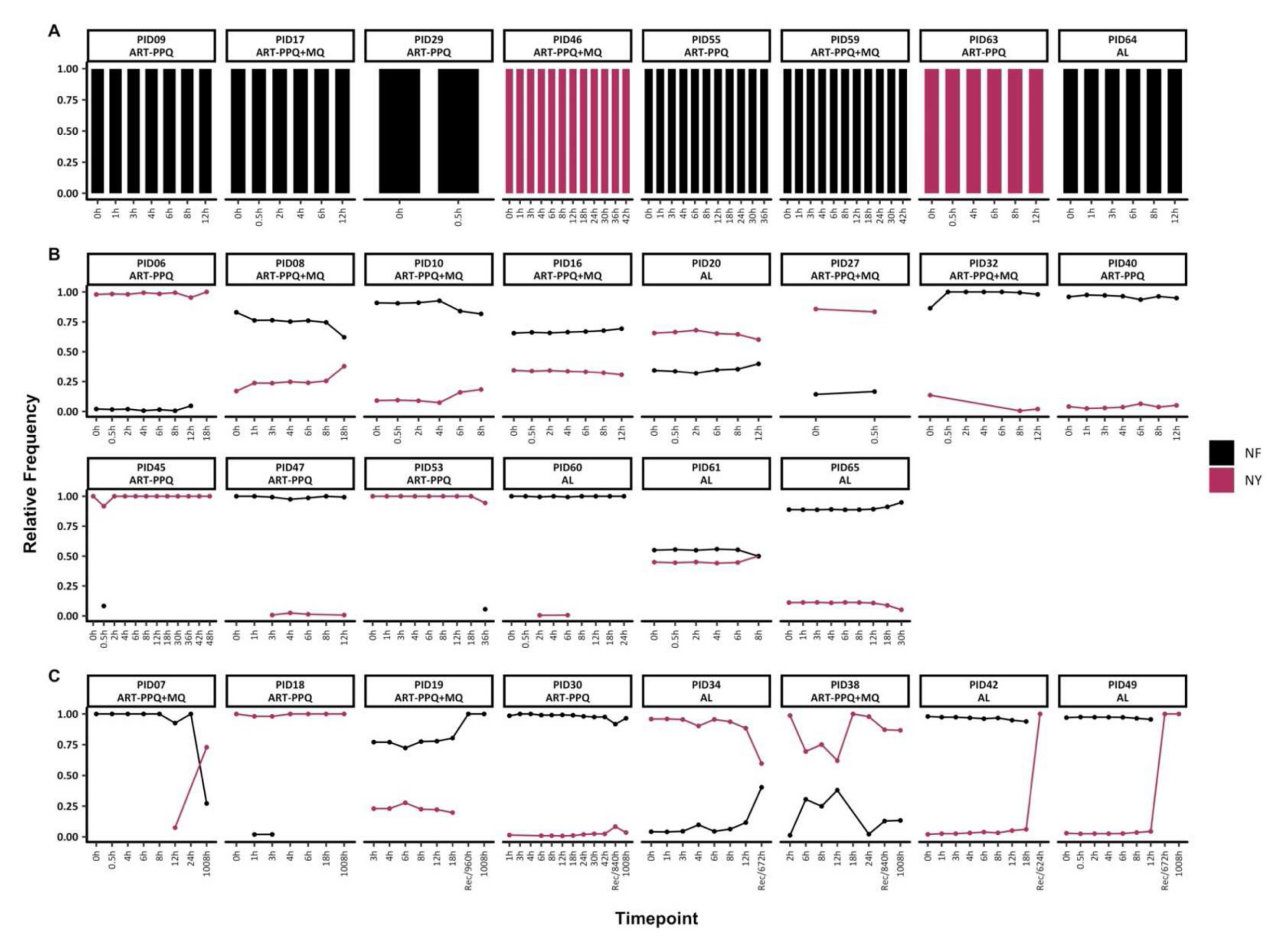
Temporal changes in *mdr1* microhaplotypes throughout treatment. The figure highlights the individuals with **A**) monoclonal infections, if they had only one mdr1 at any timepoint **B**) polyclonal infections, if they had more than one mdr1 microhaplotype throughout treatment and sampled up to 72h and **C**) polyclonal infections, if they more than one mdr1 microhaplotype throughout treatment and sampled beyond 72h. Each coloured barplot/point/line represents a unique mdr1 microhaplotype (black-NF and maroon-NY). Above each plot is the respective participant id. The x-axis represents the sampling timepoints at different hours and the recurrence time with the prefix "Rec" while the y-axis represents the relative proportions of mdr1 microhaplotypes.

Based on the combination of amino acid polymorphisms at codons N86Y and F184Y, only two microhaplotypes (NY and NF) were detected. Of the 30 individuals with baseline data, 22 had mixed infections with both mdr1 microhaplotypes, while 8 had monoclonal *mdr1* infections (either NF or NY) throughout treatment ( Figure 3A). Of the individuals with mixed *mdr1* infections, 15/22 had sequence data up to 72h only, and they maintained *mdr1* microhaplotypes at stable frequencies, similar to *ama1*. However, in two individuals (PIDs 45 and 53), there were sporadic detections of rare *mdr1* microhaplotypes ( Figure 3B).

In the remaining 8/22 individuals with mixed *mdr1* infections and with post-treatment (>72h) sequence data, three of these individuals switched from a predominance of the NF microhaplotype to the NY microhaplotype during follow-up either during recurrence infection (unscheduled visit) or on day 42 (1008h). Only PID18 maintained the same dominant microhaplotype (NY) throughout the treatment and follow-up period on day 42 (1008h) ( Figure 3C). There was no significant difference in the frequencies of Y184 and 184F microhaplotypes in baseline samples vs samples collected from 12h onward (Chi-square test, *p* = 0.52).

## Discussion

Children in this moderate-high malaria transmission setting in Kilifi maintained a stable homogeneous microhaplotype population throughout treatment until day 3 (72h). This is not surprising since febrile infections tend to occur with low COI even with high parasite densities ( Beck *et al.*, 1997) and treatment reduces the establishment of new infections. Thus, the genetic homogeneity improves the confidence in determining reinfections post-treatment (day 28 onwards), such as the one individual identified with a microhaplotype at 30h and later in their recurrent infection (for an unscheduled visit at 672h). The sporadic observation of rare microhaplotypes at only a single time point potentially highlights the changes in parasite density, sequestration of parasites, lingering genetic material from dead parasites or the presence of parasites below the blood sampling limit ( Jones *et al.*, 2021). Notably, PID27 had a COI of three at 0h, and while the relative frequencies of two *ama1* microhaplotypes decreased over time, one microhaplotype continued to increase in frequency. This patient did not experience a recurrence and had no microscopically detectable parasites by 72h. Therefore, the increase in one *ama1* microhaplotype may have originated from the nucleic material of dead parasites. Most of these distinct changes in *ama1* microhaplotype frequencies occurred post-treatment from day 7 onwards, when reinfections are likely as the levels of drugs in the body continue to decrease. Importantly, our analysis of rare microhaplotypes was limited to a 5% cut-off based on sequencing controls to increase our confidence in calling mixed infections.

The clearance rates were similar within and between individuals irrespective of clonality, suggesting that the three antimalarial treatments were equally effective in clearing microhaplotypes and parasitaemia. Any significant deviations in microhaplotype clearance rates would signal emerging resistance during treatment if a microhaplotype was consistently cleared at a slower rate within and between individuals. This analysis identified one such individual with an estimated slow clearance of 5.7h. A closer examination of the two main microhaplotypes in this participant identified a slow clearing microhaplotype with an estimated clearance half-life of 7h. Thus, a drug-resistant microhaplotype circulating at a low frequency before treatment may survive and rapidly expand following treatment ( Ecker *et al.*, 2012; Jafari *et al.*, 2004). In addition to the slow clearing microhaplotype, the sole fast and three slower clearing microhaplotypes indicate the variation in an individual’s ability to clear an infection. Such infections should be interrogated to determine additional factors contributing to the slow clearing of parasites.

The *mdr1* genotype in codon 86 was 100% wild type (N86). This is consistent with previous findings ( Wamae *et al.*, 2019) in the study area of a shift from 86Y to N86 by 2018 when this study began, and thus only two microhaplotypes were observed based on codon 184. Though the sample size was small, as expected, there was no selection of codon 184 with these drugs. This is in contrast to a study conducted in Tanzania between 2002 and 2004, when there was a higher frequency of *mdr1* mutant genotypes, that observed more N86 and 184F in post-treatment samples following artemether-lumefantrine treatment ( Humphreys *et al.*, 2007).

Limitations of this study include the small sample size across the three treatment arms, which may have led to biases. For example, all but one of the recurrent infections were new infections with entirely different microhaplotypes from the pre-treatment sample by *ama1* AmpSeq. Therefore , in more extensive studies, it remains to be seen how sensitive AmpSeq will be in distinguishing new vs recurrent infections compared to msp1/mps2/glurp genotyping. The observations made from a single individual with a slow clearing microhaplotype require further validation as this was based on data from only one individual. Moreover, since this study was set up to provide a proof of concept, it is a scalable assay that allows for additional drug resistance markers, such as *k13*, to be monitored. The low parasitaemia following treatment minimised the generation of good quality AmpSeq data, impacting the sample size. Consequently, there were limited samples to examine the changes in microhaplotypes throughout the study period. However, the findings were similar to several drug trials that predominantly identified new rather than recrudescent infections when efficacious drugs are tested ( Adegbite *et al.*, 2019; Davlantes *et al.*, 2018; Kakolwa *et al.*, 2018). Additionally, immunity may play a role in clearing infections; thus, new microhaplotypes are likely present in subsequent infections. Microhaplotypes detected post-treatment might have originated from circulating gametocytes, dormant or dead parasites. However, all participants were gametocyte negative throughout treatment except PID38 who had five gametocytes/μl at 72h but was negative thereafter, so it is unlikely that gametocytaemia biased our findings. As for genetic material originating from dormant or dead parasites, parasite mRNA can also be detected up to two weeks after successful treatment in microscopy-negative individuals ( Mahamar *et al.*, 2021). However, additional work is needed to determine whether these originate from viable infections. Finally, future studies should include artificial sequencing controls of decreasing parasitaemia from, e.g., dilutions ranging from 10,000 to 1 parasite/μl to reliably determine the limit of detection, especially in post-treatment with low parasitemia.

Improved accuracy in distinguishing infections post-treatment is essential when considering the WHO recommendation of abandoning a drug if failure rates are >10% ( WHO, 2008), determined by the number of recrudescent infections that is likely to vary based on genotyping methods used and genetic markers examined. The additional analyses of tracking variants throughout treatment improve the ability to identify dominant pre-treatment variants that appear as a new infection (in the follow-up period) and are misclassified as a recrudescent infection. *Ama1* yielded comparable COI to *msp1* and *msp2*, hence, the *ama1* locus genotyped in this study is a highly discriminatory marker with a larger number of variants at a prevalence of <5% and with a high expected heterozygosity value (0.96). Coupled with the high sensitivity of amplicon sequencing to capture minor variants, this assay provides a higher resolution to better distinguish reinfections from recrudescent infections in moderate to high transmission settings where polyclonal infections are common.

The assessment of genotypes throughout treatment follows the trajectory of infection, identifies the number of infecting microhaplotypes and rare variants per individual. This allows for the early detection of emerging resistant variants by identifying slow-clearing variants. Furthermore, examining drug resistance mutation frequencies during treatment can identify distinct shifts in occurrences likely to indicate directional selection of a rapidly rising variant ( Henriques *et al.*, 2014; Mideo *et al.*, 2013). Given the high disease burden and high levels of reinfection in sub-Saharan Africa, there is a need for improved tools for conducting molecular assays to improve the interpretation of the genotyping outputs. In fact, a recent WHO consultation meeting concluded that AmpSeq provides the most robust and reliable genotyping method ( WHO, 2021b). The AmpSeq assay, presented in this study, highlights a potential genotyping tool for PCR correction and monitoring drug resistance markers. The need for more genotyping reference labs in Africa remains to support this endeavour.

## Data Availability

The raw fastq files have been deposited in Zenodo under: (Fastq Files) Amplicon sequencing of *ama1* and *mdr1* to track within-host P. falciparum diversity in Kilifi, KENYA (Version 1) [Data set]. Zenodo. https://doi.org/10.5281/zenodo.6243929 The nucleotide sequence data reported in this paper are available in the GenBank database under the accession numbers: *ama1* (MZ593448 - MZ593480) and *mdr1* (MZ593481 - MZ593484). Data are available under the terms of the Creative Commons Attribution 4.0 International license (CC-BY 4.0). Extended data tables and figures have been deposited in Zenodo under: (Extended Data) Amplicon sequencing of ama1 and mdr1 to track within-host P. falciparum diversity throughout treatment in a clinical drug trial (Version 1) [Data set]. DOI: https://doi.org/10.5281/zenodo.6253570 (Wamae *et al.*, 2022b). This collection contains the following extended data: **Table S1. List of PCR and deep sequencing primers**. This table shows the list of forward and reverse primers used for deep sequencing. In boldface are the MID tags, while in the regular face are the forward primers **Table S2. The relative frequencies of each ama1 variant and the number of samples with each variant** *.* The relative frequencies (%) of the 33 AMA1 variants in pre-and post-treatment samples (n = 330) are shown as a 33 amino acid sequence. The frequencies were calculated by dividing the number of reads of each microhaplotype by the total number of reads obtained per sample (116,187,131) **Table S3. The parasitemia levels associated with each *ama1* microhaplotypes per timepoint** *.* This table shows the parasitemia for each *ama1* microhaplotype per timepoint and each participant. “Patient ID” represents the patient ID, “AMA1 COI at 0h” represents the complexity of infection (COI) for each participant at baseline, based on *ama1* while subsequent columns represent the parasitemia for each *ama1* microhaplotype from timepoint 0h to 1008h. Parasitemia was back-calculated using the COI and total parasitemia for each timepoint. For time points with a COI > 1, parasitemia for the respective *ama1* microhaplotypes are separated by commas, cells in red indicate timepoints without sequencing data (ND = not determined). In contrast, cells in grey indicate timepoints where microhaplotypes were detected below 10 parasites/μl, hence at risk of falling below the sampling limit. **Figure S1. Performance of AmpSeq in the sequencing controls.** Six replicates were prepared for each control set to ensure sufficient control data in case of PCR or sequencing failure. The median read depth in the lab controls was 5658 (range 4,310 – 12,603) and 658 (- 1,676). The x-axis represents the replicate identifier across the five mixtures, starting from 1 to 6, while the y-axis represents the proportions of each variant across all replicates. For *ama1* (A), two variants (3D7 and Dd2) were detected, whereas in *mdr1* (B), two variants were detected YY, FY and NY following amplification of Dd2 Copy I, Dd2 Copy II and 3D7, respectively. For *ama1*, sequencing failed for replicate 6 of control set 1, while for *mdr1*, sequencing failed for replicate 2 and 6 of control set 3, replicates 1 and 6 of control set 4 and replicates 1 and 5 of control set 5. Under the *mdr1* control set 4, the Dd2 copy II (86F, 184Y) was not identified, possibly due to having very low concentrations that were not picked up in this replicate. Based on our control mixtures, the minimum variant frequency we could detect was 0.5%. **Figure S2. Heatmaps of the successfully PCR amplified and sequenced samples for *ama1* (A) and *mdr1* (B).** The rows represent the study participants, while the columns represent time in hours. Successfully sequenced samples are shown in blue, those that failed PCR are shown in red and those that failed sequencing are in black. The timepoint “ *Rec*” represents unscheduled visits where a recurrent sample was collected. The unshaded areas with "-" are time points where samples were not collected. For each time-point, the number of samples successfully sequenced (n), is indicated in the last row of each panel. The table in panel C shows the groupings of samples based on parasitemia, high (> 5,000), moderate (100-5,000) and low (< 100 parasites per microlitre). Many samples collected between 0h-12h had high parasitemia, samples collected between 18h-30h had moderate parasitemia, while samples collected after 30h were primarily of low parasitemia. **Figure S3. The mean complexity of infection (COI) by AMA1 throughout treatment.** The mean COI (red diamonds) appeared to be stable (between 1.5 - 2) from baseline (0h) up to 72h and thereafter fluctuated due to the small sample sizes (<5) in the post-treatment samples. The black dots represent the COI per sample. Data are available under the terms of the Creative Commons Attribution 4.0 International license (CC-BY 4.0).
