# Supplementary material for: Targeted amplicon deep sequencing of ama1 and mdr1 to track within-host P. falciparum diversity throughout treatment in a clinical drug trial
Source: Wellcome Open Res. 2024 Mar 14;7:95. Originally published 2022 Mar 16. [Version 4] doi: 10.12688/wellcomeopenres.17736.4 (PMC10349275; doi:10.12688/wellcomeopenres.17736.4)
Supplement: Peer review history

# Reviewer report: Reviewer response for version 2

**DOI:** 10.21956/wellcomeopenres.20663.r61598

**Published:** 2023-07-25

**Authors:** Aurel Holzschuh

**Affiliations:** 1 University of Notre Dame, Notre Dame, Indiana, USA

In this study, the researchers investigated the potential of AmpSeq for monitoring the within-host diversity of parasites before and after ACT treatment. They examined the presence or absence of *ama1 *and *mdr1 *microhaplotypes in blood samples collected at regular intervals from patients treated with three different regimens. This study shows the potential of AmpSeq as a tool for tracking parasite microhaplotypes throughout treatment. However, I think that there is some clarification needed regarding the methods and results. I have some comments for the authors to address:

**Methods:**

*1. DNA preparation and PCR from sequencing controls and clinical samples*

 The authors noted that they could detect the minority allele in a mixture of 95%:5% and regarded any reads less than 5% of the total as noise. However, I could not find any information regarding the parasite densities of the artificial control mixtures. I assume the different ratios were all at the same total parasite density? Reliable detection of a 5% minority clone at e.g., 1,000 parasites/uL (i.e., 50 parasites/uL at 5%) does not guarantee detection of a 5% minority clone at 100 or 10 parasites/uL. It seems like the authors have chosen to include all samples with a “blood sampling limit” of ≥10 parasites/uL, do you have any control that can confirm the detection of 5% minority clones at this parasite density? If you look at your control mixtures, what are the number of reads for those? In control set 4, Rep_4, marker mdr1, did you obtain fewer reads for this sample and thus missed the YY microhaplotype (maybe it was only below the 5% threshold)?

*2. DNA preparation and PCR from sequencing controls and clinical samples*

 Regarding the sequencing controls, you mention the following: “Amplicons spanning *ama1* (PF3D7_1133400, nucleotides 441–946) and *mdr1* (PF3D7_0523000, nucleotides 183–719) were generated in duplicate from each control and sample,…”. However, Figure S1 indicates 6 replicates for each control set. Under sequence data analysis you again mention: “Microhaplotypes were discarded if they did not occur in the two PCR replicates and if their combined relative frequency was <5%.” Please specify.

*3. Sequence data analysis*

 Was there a minimum number of reads needed to call a microhaplotype, e.g., 10 reads? The lower the total reads for a sample, the more I expect that whether or not certain microhaplotypes were detected was a matter of chance, especially for low-abundant minority clones. As an example, replicate 1 has 1,000 total reads and two clones, one with 960 reads and the second with 40 reads. You would discard the minor clone because of the >5% threshold. Replicate 2 has 500 total reads and also two clones. One with 460 reads and the second with 40 reads. You would consider this a polyclonal infection because the minor clone is above 5%. Could you give a brief explanation if only the 5% threshold was considered to call a microhaplotype or if the number of reads was also considered?

**Results:**

*4. AmpSeq in artificial mixtures of sequencing controls*

 The authors state: “This provided evidence that the assay could detect mixed infections in clinical samples, but only when the minor microhaplotype was at a relative frequency of ≥5%.” This is very much density-dependent. What was the parasite density of the controls? Please add the density of the controls, as evidence is only there for the parasite densities used (which was?). With decreasing parasitemia, the likelihood of detecting minority clones at 5% also declines (becomes a chance event at one point).

 Figure S1. Control set 2 and 3 are either mixed up, or the observed proportions do not really match the expected proportions. In the results section the authors state “…the 3D7 and Dd2 *ama1* microhaplotypes were detected consistently across all mixtures and in the expected proportions.” Looking at control set 2, the minority clone seems to be around 25%, and not at the expected 15%. Similarly, for control set 3, the minority clone is around 15% whereas it would be expected at around 15%. Please check.

 Figure S1. In the legend, the authors state “Based on our control mixtures, the minimum variant frequency we were able to detect was 0.5%.”. I think this should be 5% and not 0.5%.

*5. AmpSeq of pre-and post-treatment samples*

 Figure S2. The legend states “Successfully sequenced samples are shown in green, those that failed PCR in red, and those that failed sequencing are in yellow.” I do not see any green or yellow color in the heatmap, I see blue, red, and black. Please correct. Similarly, the authors state “For each time-point, the number of samples successfully sequenced, *n*, is indicated in the last row of each panel”. I do not see any *n* in either of the panels.

*6. Pfama1 genetic diversity during and after treatment*

 Figure 1. Could the authors maybe provide the parasite densities for each sample at each time point instead of only stating high and low? This could explain some of the rare haplotypes popping up potentially. Where rare haplotypes occurred, did the authors see if the same rare haplotype was detected at other time points but possibly below the 5% threshold (check if reads were present)? Was there a difference in the number of reads between the monoclonal samples and polyclonal samples?

 Maybe it would be good if some of those sporadic microhaplotypes were present but below either the 5% BIC or the 10 parasites/μl blood sampling limit (could also be provided as supplementary information).

 Table S2. All 33 ama1 variants reported seem to be nonsynonymous. Is it true that the authors only observed nonsynonymous mutations in the *ama1* microhaplotype leading to 33 different microhaplotypes at the AA level? What about SNPs that are synonymous? In the Methods section under “Sequence data analysis” you state the following: “COI was defined as the number of distinct *ama1* microhaplotypes (varying at the nucleotide level) in each sample”.

 Except for PID60, all these sporadic microhaplotypes were detected above the sampling limit of 10 parasites/μl (Table S3). So why is this sporadic microhaplotype shown for PID60 if it fell below the 10 parasites/μl? In the methods section you specify this threshold, thus should be removed. Were there other sporadic microhaplotypes that fell below the 10 parasites/ul threshold but were excluded?

*7. Parasite clearance estimates*

 “Participant PID32 had one ama1 microhaplotype, V9, that was cleared quite rapidly at <1h…”. Looking at Figure 1B, I do not see any microhaplotype that was cleared <1h. I only see a microhaplotype (light blue) that persists throughout and a sporadic microhaplotype at 8h). Am I missing something here? Without any parasite densities for the samples/time points, it’s impossible to tell based on the proportions of Figure 1 (as we do not see any drop in parasitemia if only the proportions of each microhaplotype are shown). Again, consider adding this information somewhere.

*8. AmpSeq compared to msp1/msp2/glurp genotyping of recurrent infections*

 Might be helpful to also calculate expected heterozygosity for *msp1*/ *msp2*/ *glurp *since you provide this metric for *ama1* and mention in the discussion “the *ama1* locus genotyped in this study is a highly discriminatory marker”.

*9. Pfmdr1 genetic diversity pre-and post-treatment*

 Interestingly, some of the COI based on ama1 and mdr1 are not concordant. PID06, PID07, PID18, PID42, PID47, and PID53 are all monoclonal based on *ama1* but polyclonal based on *mdr1*. I would expect the other way around, where several different ama1 microhaplotypes likely share the same mdr1 microhaplotype. For most of them, the mdr1 minor microhaplotype is present at very low proportions. PID42 is somewhat concordant (when the 2 ^nd^ clone takes over at 642h) as you detect the second clone but miss the first clone (possibly just below the 5% threshold?). Any explanation for this? Maybe take a closer look at the blood sampling limit or the 5% BIC? *ama1* generally seemed to have worked better (e.g., generated a higher number of reads and higher sequencing success).

Is the work clearly and accurately presented and does it cite the current literature?

Yes

If applicable, is the statistical analysis and its interpretation appropriate?

Yes

Are all the source data underlying the results available to ensure full reproducibility?

Yes

Is the study design appropriate and is the work technically sound?

Partly

Are the conclusions drawn adequately supported by the results?

Partly

Are sufficient details of methods and analysis provided to allow replication by others?

Partly

Reviewer Expertise:

Molecular diagnostics and genomic epidemiology of malaria.

I confirm that I have read this submission and believe that I have an appropriate level of expertise to confirm that it is of an acceptable scientific standard, however I have significant reservations, as outlined above.

# Reviewer report: Reviewer response for version 2

**DOI:** 10.21956/wellcomeopenres.20663.r61601

**Published:** 2023-07-14

**Authors:** Eniyou Cheryll Oriero

**Affiliations:** 1 Medical Research Council Unit The Gambia at LSHTM, Fajara, The Gambia

The manuscript evaluated the use of amplicon sequencing to simultaneously determine complexity of *Plasmodium falciparum* infection, PCR correction and drug resistance following administration of triple combination ACTs. There are a few minor comments for the authors to address:

1. Parasite clearance: The authors have shown half-life graphs of parasite clearance estimates, adding a kaplan-meier curve of parasite clearance by microscopy and PCR (if data is available) will support parasite genotyping at the 72hr time point post-treatment, with evidence of PCR detectable parasites at that time point.
2. Though the manuscript is a proof-of-concept study with added advantage of simultaneous drug resistance genotyping, it has not sufficiently shown superiority of AmpSeq over *msp1/msp2/glurp* genotyping for PCR correction and determining complexity of infection. The authors may want to comment further on the cost-effectiveness and availability of expertise (e.g bioinformatics) for AmpSeq in LMICs.
3. The authors used artificial mixtures of two parasite strains to generate sequencing controls with artificial microhaplotypes similar to natural mixed infections. The authors should comment if they were able to determine copy number variation (particularly for *mdr1*) using read counts obtained from the AmpSeq data.

Is the work clearly and accurately presented and does it cite the current literature?

Yes

If applicable, is the statistical analysis and its interpretation appropriate?

Yes

Are all the source data underlying the results available to ensure full reproducibility?

Yes

Is the study design appropriate and is the work technically sound?

Yes

Are the conclusions drawn adequately supported by the results?

Yes

Are sufficient details of methods and analysis provided to allow replication by others?

Yes

Reviewer Expertise:

Molecular diagnostics, Genomic epidemiology, non-falciparum malaria

I confirm that I have read this submission and believe that I have an appropriate level of expertise to confirm that it is of an acceptable scientific standard.

# Response

**Published:** 2023-09-22

**Authors:** Kevin Wamae

**Affiliations:** KEMRI-Wellcome Trust Research Programme, Kenya

***We want to thank Dr. Eniyou for agreeing to review this article, and we respond to her questions below:***

1. ***Parasite clearance: The authors have shown half-life graphs of parasite clearance estimates, adding a Kaplan-Meier curve of parasite clearance by microscopy and PCR (if data is available) will support parasite genotyping at the 72hr time point post-treatment, with evidence of PCR detectable parasites at that time point: ***
  The information presented in Figure S2C demonstrates that all individuals had eliminated the parasites by day 7 (hour 168). Therefore, the Kaplan-Meier (KM) analysis does not give us more information beyond this and is better suited to the parent study that included a larger sample size to conduct the KM analysis. It's noteworthy that parasites were undetectable by microscopy beyond 72 hours, and we acknowledge that we encountered challenges with PCR and sequencing failures from this point onward, which we discussed as one of the limitations in our study's discussion section. 
  “ *…The low parasitaemia following treatment minimised the generation of good quality AmpSeq data, impacting the sample size. Consequently, there were limited samples to examine the changes in microhaplotypes throughout the study period…*”  
2. **Though the manuscript is a proof-of-concept study with added advantage of simultaneous drug resistance genotyping, it has not sufficiently shown superiority of AmpSeq over msp1/msp2/glurp genotyping for PCR correction and determining complexity of infection. The authors may want to comment further on the cost-effectiveness and availability of expertise (e.g. bioinformatics) for AmpSeq in LMICs:** 
  ​​​​​​​​​​​​​​We have amended the manuscript to acknowledge this observation in the last three paragraphs of the discussion, as indicated below:  
*“...While this study serves as a proof of concept, the sample size employed was not aimed at establishing the clear superiority of AmpSeq over msp1/msp2/glurp genotyping for tasks such as PCR correction and determining infection complexity. Moreover, the adoption of AmpSeq in low and middle-income countries (LMICs) is challenged by its current cost and the requisite molecular biology and bioinformatics expertise needed for effective implementation. Nevertheless, the constantly declining costs of next-generation sequencing, coupled with emerging technologies like the Oxford Nanopore platform that offer reduced buy-in and maintenance expenses, hint at a potentially more accessible AmpSeq in the future.* *Regarding its performance, our study illustrates that AmpSeq yields results comparable to those obtained from msp1/msp2/glurp genotyping. However, AmpSeq boasts additional benefits over msp1/mps2/glurp genotyping. It demonstrates scalability advantages and heightened resolution based on sequence identity, surpassing the limitations of msp1/msp2/glurp genotyping, such as the reliance on labour-intensive gel electrophoresis-based band size analysis or fragment analysis that can be subject to interpretation errors and the inability to detect low-density parasite clones. An additional AmpSeq's strength lies in its potential for multiplexing, enabling the simultaneous identification of drug resistance markers alongside genotyping. This multiplexing capability enhances the assay's versatility and could offer valuable insights into the parasite's drug resistance profiles.* *In light of these considerations, while the current prerequisites of expertise and cost might impede the immediate integration of AmpSeq in LMICs, the evolving landscape of next-generation sequencing technologies and cost reductions inspire optimism regarding AmpSeq's future accessibility. Therefore, this study* *recognises* *the prospective value and adaptability of AmpSeq for tasks like PCR correction and determining infection complexity, even though broader adoption may necessitate ongoing advancements in affordability and the availability of expertise…”*    
3. **The authors used artificial mixtures of two parasite strains to generate sequencing controls with artificial microhaplotypes similar to natural mixed infections. The authors should comment if they were able to determine copy number variation (particularly for mdr1) using read counts obtained from the AmpSeq data:** 
  ​​​​​​​In our study, we employed AmpSeq, a method characterised by deep sequencing of targeted regions but with shorter read lengths than whole-genome sequencing. While AmpSeq excels in the depth of sequencing within specified zones, the comprehensive detection of CNVs demands a wider genomic scan to pinpoint duplications or deletions. Concerning the *mdr1* gene, our sequencing strategy was not tailored for CNV identification. As such, while AmpSeq might provide preliminary indications of CNVs through read-depth analysis, robust and precise CNV assessments necessitate other techniques, and would require the inclusion of controls that include mdr1 genes with CNVs for comparison. This was not an objective of our study, and we primarily focused on characterising microhaplotypes for drug resistance.

# Reviewer report: Reviewer response for version 2

**DOI:** 10.21956/wellcomeopenres.20663.r53729

**Published:** 2023-01-20

**Authors:** Mita Toshihiro

**Affiliations:** 1 Department of Tropical Medicine and Parasitology, Juntendo University, Tokyo, Japan

I read the detailed response and finally found that revision was satisfactory.

Is the work clearly and accurately presented and does it cite the current literature?

Partly

If applicable, is the statistical analysis and its interpretation appropriate?

Not applicable

Are all the source data underlying the results available to ensure full reproducibility?

Yes

Is the study design appropriate and is the work technically sound?

Yes

Are the conclusions drawn adequately supported by the results?

Partly

Are sufficient details of methods and analysis provided to allow replication by others?

Partly

Reviewer Expertise:

NA

I confirm that I have read this submission and believe that I have an appropriate level of expertise to confirm that it is of an acceptable scientific standard.

# Reviewer report: Reviewer response for version 1

**DOI:** 10.21956/wellcomeopenres.19627.r49308

**Published:** 2022-05-31

**Authors:** Mita Toshihiro

**Affiliations:** 1 Department of Tropical Medicine and Parasitology, Juntendo University, Tokyo, Japan

The authors investigated the usefulness of Targeted amplicon deep sequencing (TADS) as a molecular marker to track within-host parasite diversity during and after the ACT treatments. They constructed ama1 haplotypes based on the deep sequence data and evaluated the presence/absence of haplotypes in the blood samples frequently obtained from treated patients using three different regimens. Despite that sample numbers were not so large, this study showed important findings.

 Major

1. Since dead-parasites’ DNA can persist in the human blood after treatment, the evaluate amount (also presence) of haplotypes may not have directly reflected the real situation. This may become a potential confounding factor. Could you comment on this?
2. Could you discuss the potential artifact of parasite DNA from persistent gametocytes after treatment?
3. In PID27, one clone overtook the other two clones and became dominant 6 hour after treatment. This clone has been regarded as slow clearing haplotype in the manuscript. What did the authors think the cause of slow clearing? I am interested in the discussion about this observation. I think there is a possibility that DNA from dead-sequestered parasites may have affected the result. If a particular clone tends to sequester and that DNA of such clone releases into blood stream after treatment, it will be expected that relative frequency of such parasite increases after treatment. I feel that, from your data, these may be unlikely to occur in the studied subject except from PID 27.
4. Minor haplotypes<5% were excluded from the analysis in this study. This cut-off was set based on “the lowest sequencing control’s mixture (95% 3D7 vs 5% Dd2)”. This is a bit unclear for me because next to this control mixture was 0% vs 100%. If control mixture of 99% 3D7 and 1% Dd2 shows almost same ratio in the TADS, will the authors take this cut-off?
5. In the abstract, they described that “This study highlights TADS as a sensitive tool for tracking parasite haplotypes throughout treatment ~”. However, it might be difficult to say “sensitive tool” without detection limit of this deep-sequencing method. Furthermore, I am very interested in whether TADS is relatively useful compared to traditional methods in terms of sensitivity. However, I could not find the data that compared the parasite detection ability between TADS and molecular methods (msp-1, msp-2 and glurp) recommended by WHO. Would it be possible to show further data?

Minor

1. Page 4; “Nonetheless, artemisinin (ART) resistance emerged and spread in Southeast (SE) Asia, evidenced as delayed parasite clearance following ACT treatment.” Emergence of artemisinin resistance has been already evidenced in our study in Northern Uganda (Balaikagara et al. NEJM 2021) ^1^ and Rwanda (Uwimana et al. Lancet Infect Dis, 2021). ^2^ These can be included in the Introduction.
2. Page 4; “The DNA from 3D7 and Dd2, were mixed as follows to come up with sequencing controls: 100%:100%, 75%:25%, 85%:15%, 95%:5% and 100%:0% to determine the lowest limit of haplo-type detection.”  “Does “100%:100%” mean “50%:50%”?
3. Page 4; How much blood volume did you use for deep sequencing?
4. Page 5; The word “haplotype” is defined as “The paired consensus reads for each sample were trimmed and clustered to estimate the frequency of clusters (henceforth referred to as “haplotypes”)”. However, “haplotype” was also used when it implies parasite clone, e.g. “TADS can also identify slow clearing haplotypes, a potential early sign of selection during treatment.” I understand that strikingly they are not clones, but haplotypes used here are different from the aforementioned definition.
5. Page 5; “There was successful detection of the two expected ama1 haplotypes from the 3D7 and Dd2 laboratory isolates, as well as three mdr1 haplotypes, one from 3D7 and the 2 copies of Dd2”. I think some readers might feel curious about two haplotypes in the laboratory clone (Dd2). Could add some explanation?
6. Page 6; “There was only one exception as individual PID42 had data post-72h and there was a change in the ama1 haplotype detected before and after 72h.” How did you interpret this case?
7. Figure S1; There are inconsistency of color between the figure and legend. It seems to be successfully sequenced samples in blue and failed sequencing in black. Furthermore, I could not find the number of samples successfully sequenced in the last row in each panel.

Is the work clearly and accurately presented and does it cite the current literature?

Partly

If applicable, is the statistical analysis and its interpretation appropriate?

Not applicable

Are all the source data underlying the results available to ensure full reproducibility?

Yes

Is the study design appropriate and is the work technically sound?

Yes

Are the conclusions drawn adequately supported by the results?

Partly

Are sufficient details of methods and analysis provided to allow replication by others?

Partly

Reviewer Expertise:

Malaria drug resistance

I confirm that I have read this submission and believe that I have an appropriate level of expertise to confirm that it is of an acceptable scientific standard, however I have significant reservations, as outlined above.

## References

[1] : Evidence of Artemisinin-Resistant Malaria in Africa. *New England Journal of Medicine* .2021;385(13) : 10.1056/NEJMoa2101746 1163-1171 10.1056/NEJMoa2101746 34551228

[2] : Association of Plasmodium falciparum kelch13 R561H genotypes with delayed parasite clearance in Rwanda: an open-label, single-arm, multicentre, therapeutic efficacy study. *The Lancet Infectious Diseases* .2021;21(8) : 10.1016/S1473-3099(21)00142-0 1120-1128 10.1016/S1473-3099(21)00142-0 33864801 PMC10202849

# Response

**Published:** 2022-09-27

**Authors:** Kevin Wamae

**Affiliations:** KEMRI-Wellcome Trust Research Programme, Kenya

Major

 1. Since dead-parasites’ DNA can persist in the human blood after treatment, the evaluate amount (also presence) of haplotypes may not have directly reflected the real situation. This may become a potential confounding factor. Could you comment on this? *We respond to this under point 2.*

****

 2. Could you discuss the potential artifact of parasite DNA from persistent gametocytes after treatment?

- *It’s possible that some of the signals detected originated from gametocytes, dead or dormant parasites and we have included this as one of the study’s limitations in the discussion:*
***“Microhaplotypes detected post-treatment might have originated from circulating gametocytes, dormant or dead parasites. However, all participants were gametocyte negative throughout treatment except PID38 who had five gametocytes/μl at 72h but was negative thereafter, so it is unlikely that gametocytaemia biased our findings. As for genetic material originating from dormant or dead parasites, parasite mRNA can also be detected up to two weeks after successful treatment in microscopy-negative individuals (Mahamar et al. 2021). However, additional work is needed to determine whether these originate from viable infections”***

3. In PID27, one clone overtook the other two clones and became dominant 6 hour after treatment. This clone has been regarded as slow clearing haplotype in the manuscript. What did the authors think the cause of slow clearing? I am interested in the discussion about this observation. I think there is a possibility that DNA from dead-sequestered parasites may have affected the result. If a particular clone tends to sequester and that DNA of such clone releases into blood stream after treatment, it will be expected that relative frequency of such parasite increases after treatment. I feel that, from your data, these may be unlikely to occur in the studied subject except from PID 27.

- *We have included a sentence in the discussion to highlight this individual and we hypothesize that the increase in one ama1 haplotype may have come from DNA of dead parasites:*
***“Notably, PID27 had a COI of three at 0h, and while the relative frequencies of two ama1 microhaplotypes decreased over time, one microhaplotype continued to increase in frequency. This patient did not experience a recurrence and had no microscopically detectable parasites by 72h. Therefore, the increase in one ama1 microhaplotype may have originated from the nucleic material of dead parasites.”***

*** ***


 4. Minor haplotypes <5% were excluded from the analysis in this study. This cut-off was set based on “the lowest sequencing control’s mixture (95% 3D7 vs 5% Dd2)”. This is a bit unclear for me because next to this control mixture was 0% vs 100%. If control mixture of 99% 3D7 and 1% Dd2 shows almost same ratio in the TADS, will the authors take this cut-off?

- *The control mixture 0% vs 100% was basically a clonal mixture of 3D7 and this helped to evaluate whether we would detect false positives since we expect to find only one microhaplotype. Conversely, the control’s mixture 95% 3D7 vs 5% Dd2 was multiclonal to determine the ability of the assay to pick up multiclonal infections as well as multiclonal infections with minor variants.* 
*To respond to the last question, yes, if we included an artificial mixture of 3D7 and Dd2 with lower than 5% frequency and detected haplotypes, we would have taken that cut-off.*

In the abstract, they described that “This study highlights TADS as a sensitive tool for tracking parasite haplotypes throughout treatment ~”. However, it might be difficult to say “sensitive tool” without detection limit of this deep-sequencing method. Furthermore, I am very interested in whether TADS is relatively useful compared to traditional methods in terms of sensitivity. However, I could not find the data that compared the parasite detection ability between TADS and molecular methods (msp-1, msp-2 and glurp) recommended by WHO. Would it be possible to show further data?

  

- *Unfortunately, we do not have this data and we acknowledge this limitation in the discussion:*
***“…Finally, future studies should include artificial sequencing controls of decreasing parasitaemia from, e.g., dilutions ranging from 10,000 to 1 parasite/μl to reliably determine the limit of detection, especially in post-treatment with low parasitemia...”***

Minor

 Page 4; “Nonetheless, artemisinin (ART) resistance emerged and spread in Southeast (SE) Asia, evidenced as delayed parasite clearance following ACT treatment.” Emergence of artemisinin resistance has been already evidenced in our study in Northern Uganda (Balaikagara et al. NEJM 2021) ^1^ and Rwanda (Uwimana et al. Lancet Infect Dis, 2021). ^2^ These can be included in the Introduction.

- *We have included this in the introduction:*
***“…Two recent studies have identified early signs of ART resistance in Rwanda*** ***(Uwimana et al. 2021)*** ***and Uganda*** ***(Balikagala et al. 2021)*** ***and this looming threat of widespread ACT resistance would be catastrophic in sub-Saharan Africa, where the burden of malaria is the most significant*** ***(WHO 2021a)*** ***…”***

** **


 Page 4; “The DNA from 3D7 and Dd2, were mixed as follows to come up with sequencing controls: 100%:100%, 75%:25%, 85%:15%, 95%:5% and 100%:0% to determine the lowest limit of haplotype detection.”  “Does “100%:100%” mean “50%:50%”?

- *To make it easier for the reader to understand the mixtures, we have switched from percentages to ratios of* *0.5:0.5, 0.75:0.25, 0.85:0.15, 0.95:0.05 and 1:0.*
* *

 

 Page 4; How much blood volume did you use for deep sequencing?

- *We have included the volume in the methods:*
***“…DNA was extracted from P. falciparum laboratory reference clones, 3D7 and Dd2 (BEI Resources), and from 200μl of frozen patient blood samples using the QIAamp DNA Blood Mini Kit (Qiagen) according to the manufacturer’s instructions…” ***

****

 Page 5; The word “haplotype” is defined as “The paired consensus reads for each sample were trimmed and clustered to estimate the frequency of clusters (henceforth referred to as “haplotypes”)”. However, “haplotype” was also used when it implies parasite clone, e.g. “TADS can also identify slow clearing haplotypes, a potential early sign of selection during treatment.” I understand that strikingly they are not clones, but haplotypes used here are different from the aforementioned definition.

- *We acknowledge how confusing this was. Hence, we have clarified the use of “haplotype” by settling on the term “microhaplotype” to mean the set of amino acid polymorphisms found on a single sequence.*

  Page 5; “There was successful detection of the two expected ama1 haplotypes from the 3D7 and Dd2 laboratory isolates, as well as three mdr1 haplotypes, one from 3D7 and the 2 copies of Dd2”. I think some readers might feel curious about two haplotypes in the laboratory clone (Dd2). Could add some explanation?

- We have included the following statement in the methods:
- ***“…The sequencing controls allowed for the detection of two ama1 (3D7 and Dd2) and three mdr1 (one from 3D7 and two from Dd2) microhaplotypes. Dd2 contained two mdr1 gene copies generated due to adaptation to in vitro culture…”***

  Page 6; “There was only one exception as individual PID42 had data post-72h and there was a change in the ama1 haplotype detected before and after 72h.” How did you interpret this case?

- *To present the data on changes of ama1 microhaplotypes throughout treatment, we grouped participants into those with monoclonal infections and those with multiclonal infections. PID42 had a monoclonal infection since like all individuals in this group, only one ama1 haplotype was observed per timepoint. However, unlike all monoclonal infections where the same clone at baseline was seen throughout treatment, PID42 had a change in clones at the time of recurrence. *

 
*Figure S2*; There are inconsistencies of color between the figure and legend. It seems to be successfully sequenced samples in blue and failed sequencing in black. Furthermore, I could not find the number of samples successfully sequenced in the last row in each panel.

- *Thank you for pointing out the inconsistencies in colours. We have updated the figure legend for match the* *colours* *depicted in the figure. The last row indicating the number of successfully sequenced samples was also included in Figure. S2.*

# Reviewer report: Reviewer response for version 1

**DOI:** 10.21956/wellcomeopenres.19627.r49311

**Published:** 2022-03-31

**Authors:** Ian M. Hastings

**Affiliations:** 1 Liverpool School of Tropical Medicine, Liverpool, UK

We recently published a paper investigated the use of deep sequenced amplicons in malaria drug clinical trials, in which we used extensive computer simulations of parasite dynamics post-treatment to identify how best to incorporate and interpret deep sequenced amplicon data: Jones, S., et al. (2021). ^1^

 The aim was to identify best practice for their application and to identify traps and errors likely to arise in in their initial applications. That paper was focused on molecular correction, but a prerequisite for this is good quality genotyping data and we spent a lot of time considering this.

 The authors of the current paper appear unaware of this work which is unfortunate as they appear to have fallen into some of the pitfalls we anticipate, in particular (i) the necessity of estimating a blood sampling limit (ie. the minimum number or density of parasites in a patient that is likely to be detected by the sequencing) and (ii) ability of deep sequencing to detect genetic material in circulating gametocytes. These are discussed below.

 One of our recommendations was to be very careful using the word “haplotype” as it can mean at least three distinct things in the current context (part 1 of SI in (Jones et al) ^1^

- A unique genetic variant at the amplified locus. This terminology has slipped into the bio-informatics jargon but in genetics is known as an “allele”.

- The genotype along a whole contiguous stretch of chromosome which may contain several distant alleles

- The entire malaria clonal genotype (because it is haploid in the human stages.)

So in their Abstract they use “haplotype” to mean two different things. In the first part it means allele (as in “overall *ama1 *detected more haplotypes)”. Whereas later it means the whole malaria genotype e.g. “we identified a fast (<1h) and slow (>5h) clearing haplotype.” and “clone” would be much better here than “haplotype”.

 This is not just being pedantic: using the same term to mean different thing is confusing even for authors and it is important to establish some sort of unambiguous, consensus terminology early in the development of field.

 I’ll enumerate comment for ease of cross reference.

 (1) Please be accurate and consistent with use of the term “haplotype” and consider revising the manuscript accordingly.

 (2) Note that in our publication ^1^we used the abbreviation “AmpSeq”, the current authors used “TADS” to mean the same thing. It would be nice to establish a consistent terminology noting the latest WHO recommendation also uses “AmpSeq” i.e. https://www.who.int/publications/i/item/9789240038363

 If “TADS” is already in the literature I would request (but not demand) they consider changing TADS to Ampseq. Like the use of “haplotype” it is not essential but if a field starts off by using different anacronyms for the same thing it tends to persist which is in nobody’s long-term interest. If “TADS” is not already in use in the literature it would be hard to justify  using a new terms when one already exist.

**Methods.**

 (3) I have sat through seeming interminable technical discussions on amplicon sequencing, most of which went over my head. I do know enough to realise it is not as straightforward as most people think so suggest an expert on genotyping also reviews this manuscript. The authors noted that they could detect the minority allele in mixture of 95%:5% and regarded any reads less than 5% of total as noise. We previously found this threshold to be extremely important (i.e. ^1^) and termed it the Bioinformatics cut-off (BIC), and identified it as a vital piece of information to be included in publications, so it is nice to see it clearly defined here. However, on page 8 it states “our analysis of rare haplotypes was limited to a 5% cut-off based on sequencing controls” so some clarification is needed. Is it 5% of total reads or 5% of controls? If the latter, this could be explained much more clearly.

 (4) It looks like the authors extracted their DNA from frozen blood samples. One of things we previously identified as important was the “blood sampling limit” ^1^ i.e. the number or density of parasites below which they were unlikely to be detected because the blood volume entering the genotyping assay was so small that parasites of a given genotype were statistically unlikely to be present in the sample. We made an estimate based on blood extracted from dried blood spots on filter paper. It looks like the authors need to the same calculations based on the volume of blood they used. This is important for understanding the  likely impact of gametocytes, and to interpret observed patterns (i.e. their figure 1) post treatment; see comment (10) below. They have total parasitaemia and relative frequencies of ama1 alleles in each patient so they can back-calculate likely number/density of each clone and hence figure out whether it is in danger of falling below the sampling limit. The calculations tend to be estimates but they need to do this before interpreting their figure 1 i.e. they need to be confident that all the ama1 signals in the patient are being detected and there is no danger that, at later timepoints,  one ama1 signal becoming so low (because the clone(s) containing it has fallen to very low density)  that it is no longer detected in the assay despite its continued persistence in the patient

 The blood sampling limit is also one plausible explanation for their observation (page 6) that” In five individuals (PIDs 10, 32, 40, 59 and 60), there were sporadic detections of rare *ama1 *haplotypes ” i.e. because parasitaemias of the clone containing that ama1 allele were around the sampling limit so that whether or not they were detected was a matter of chance. The second plausible reason is that it fluctuated around the 5% BIC so sporadically exceed the BIC. Discussion around these points would be good and it also emphasises why it is important to establish the sampling limit before interpreting the results

 (5) I am worried about these statements “The frequency of each haplotype in the population was calculated using the total number of samples that contained the haplotype over the total number of samples genotyped.” This will calculate prevalence *not *frequencies (this has caused immense confusion in the past). The problem can be illustrated by a simple example: assume there are only two samples: sample #1 has alleles A,B and C, and sample #2 has A, B, and D. Then according to their statement the “frequencies” of A,B, C and D will be 100%, 100%, 50% and 50% respectively which is obviously wrong as in this context the allele frequencies should sum to 100%. I assume the statement is written incorrectly and that they counted total number of clones in the two samples i.e. 3+3=6 then calculated the frequencies of A,B, C and D as 2/6. 2/6, 1/6 and 1/6 respectively i.e. ensuring frequencies sum to 100%. They then need, for reasons given below,  to calculate expected heterozygosity (He) to quantify genetic variability ie.the probably that two randomly selected clones have different alleles

 I also worry about their statement on page 10 when discussing table S2 i.e. “The frequencies were calculated by dividing the number of reads of each haplotype by the total number of reads obtained (116,187,131).” These “frequencies” are likely biased towards alleles in clones in high-parasitaemia patients. So I think the calculation of frequency suggested above is better, especially because it can be used to calculate He.

 (6) I assume the genotyping at msp1/msp2/glurp was the standard WHO-recommended method of gel electrophoresis. As above, the sensitivity (equivalent to the BIC) used in their assay should be stated. Labs typically regard genotyping peaks less than 25% or 30% of the largest peak as noise but there is no consistency in this (and some labs apparently do it “informally”) but this needs to be stated.

**Interpretation**

 (7) My main concern is that the authors seem to take no account of the possible impact of detecting alleles in gametocytes. We identified  this as a key consideration as illustrated in Figure 2 of our main text and extensively discussed in our Supplementary Material Part 3: “Simulating the potential impact of gametocytes on the accuracy of molecular correction”. ^1^  The problem is that current drugs clear the asexual form of falciparum but not the gametocytes which continue to circulate and the increased sensitivity of deep sequencing allows detection of alleles in the gametocytes. Suppose a clone initially has 1% gametocytes then after treatment their proportion increases (roughly) 2 fold with every half-life of the asexual forms i.e.  gametocytes increase (approx.) from 1% to 2% to 4% to 8% etc after 1,2,3,4, etc half-lives. The observed clearance half-life will therefore gradually decrease as the drug-insensitive gametocytes make up an increasing proportion of the clone. Whether the gametocytes will be observable in the initial test slide depends on the microscopy and on the clone size. So it’s a two-stage calculation Firstly, the extent to which any putative gametocytes will gradually reduce observed clearance rates post-treatment. Secondly, the likely impact of gametocyte signals will also depend on the blood sampling limit (i.e. whether gametocytes are of sufficient density to de detectable) which I have suggested the authors calculate (cf Fig 2 of Jones *et al*.)

 (8) First paragraph of section “Pfama1 genetic diversity during and after treatment”. The slight decrease in COI is presumably a consequence of clones dropping below the blood sampling limit (see above), or below the 5% BIC,  and being lost. Given that monoclonal infections  cannot lose diversity, it would be better to exclude these infections from the calculations to get a better idea of the drop in COI

 (9) I am extremely curious about the assertion made in the caption to figure 2 i.e. “PID32 had the fastest clearing (0.8h) *ama1 *haplotype V9.”. Looking it Figure 1, it appears that PID32 was largely monoclonal except for a single detection of another ama1 allele at time 8hours which may well be spurious. More explanation is needed.

 (10) So once background calculation have been made for blood sampling limit and impact of gametocytes there are (as far as I can see) three plausible patterns post treatment.

1. Immediate and sustained  difference in clearance rates between clones in the same patient. PID27 therefore looks the most interesting to me as is suggested significant differences in clearance between the two clones immediately post-treatment (Figure 1). Figure 2B also shows that the two clearance rates in this patient are clearly distinguishable on the Y axis unlike the other patients . Note  this could be due to differences in drug sensitivity or, less likely, to differential impact of immunity acting on the two clone.
2. An impact of gametocytes. All clones would retain roughly the same proportions immediately post-treatment, but one eventually rises. The one that increases in proportion could arise as gametocytes become a significant proportion of its biomass (see point 7 above) which are not cleared by the drug . PID08 seems to show this pattern.
3. Impact of a sampling limit. All clones maintain same proportions until the lower one falls below the sampling limit and disappears, hence the 100% has to be retained by increased proportions on those remaining above the sampling limit. This would be an artefact. Interesting, I can’t see evidence of this of Figure 1 so I’m guessing sampling limit was not a big issue (the requested background calculation should confirm this.

So these are three paradigms. There may be variation caused by factors such as sequestration,  but given that the authors chose to present and discuss the profiles, I strongly suggest they improve their discussion to include the putative/likely impact of gametocytes and sampling limits.

 (11) Page 9 they state “Hence, *ama1 *is a highly discriminatory marker with a larger number of variants with a prevalence of <5%, providing a higher resolution to better distinguish new from recrudescent infections in moderate to high transmissions settings where polyclonal infections are common.” This is probably incorrect and definitely incomplete. Msp1, msp2 and glurp are all highly variable, possible more so than ama1: they would need to report He values for ama1 to substantiate this assertion of greater genetic diversity. The most important point about deep sequencing vs msp1/msp2/glurp is sequencing has much better ability to detect minor clones… they need to cite our previous work (i..e Jones *et al*.) here as we explicitly discussed the impact of improved sensitivity in  detail.

 Recommendations.

 The manuscript is, in my opinion, methodologically and genetically naive in parts. I realize it will appear arrogant to insist they go and read our previous  investigations of this (i.e. Jones *et al*), but there was a team of us who spent a lot of time and effort anticipating likely problems (and how to mitigate them) and this is highly relevant to the work presented here.  I would recommend major revisions to the data interpretation and discussion. The underlying approach and data seem robust to me.

**Minor comments.**

 (a) Page 4. The discussion of limitations of current WHO-recommended method is a bit dated and it would be useful to cite our two recent quantitative investigations i.e.. Felger, I., et al. (2020), and Jones, S., et al. (2019). [ref-2, ref-3]

                

 Also note that WHO recently updated their genotyping recommendations to acknowledge the future importance of amplicon sequencing https://www.who.int/publications/i/item/9789240038363.

 (b) I see no point in parts (A) of figures 1 and 3…. If an infection is monoclonal the one allele will inevitable be present at 100%  i.e. the plots tell us nothing.

 (c) Page 8. The statement “The clearance rates were similar within and between individuals irrespective of clonality, suggesting that the three antimalarial treatments were equally effective in clearing haplotypes and parasitaemia.” Is a bit naive in my opinion. Differences in artemisinin sensitivity putatively result in differences in clearance rate post-treatment. However there is no suggestion this occurs for the partner drugs which mainly kill parasites when sequestered:  resistance to the partner drug will likely first show as a reduced parasite reduction ratio not as reduced clearance.

Is the work clearly and accurately presented and does it cite the current literature?

Partly

If applicable, is the statistical analysis and its interpretation appropriate?

No

Are all the source data underlying the results available to ensure full reproducibility?

Yes

Is the study design appropriate and is the work technically sound?

Yes

Are the conclusions drawn adequately supported by the results?

Partly

Are sufficient details of methods and analysis provided to allow replication by others?

Partly

Reviewer Expertise:

Malaria drug treatment and resistance.

I confirm that I have read this submission and believe that I have an appropriate level of expertise to confirm that it is of an acceptable scientific standard, however I have significant reservations, as outlined above.

## References

[1] : Should Deep-Sequenced Amplicons Become the New Gold Standard for Analyzing Malaria Drug Clinical Trials?. *Antimicrobial Agents and Chemotherapy* .2021;65(10) : 10.1128/AAC.00437-21 10.1128/AAC.00437-21 PMC844814134252299

[2] : PCR correction strategies for malaria drug trials: updates and clarifications. *The Lancet Infectious Diseases* .2020;20(1) : 10.1016/S1473-3099(19)30426-8 e20-e25 10.1016/S1473-3099(19)30426-8 31540841

[3] : Improving Methods for Analyzing Antimalarial Drug Efficacy Trials: Molecular Correction Based on Length-Polymorphic Markers msp-1, msp-2, and glurp. *Antimicrob Agents Chemother* .63(9) : 10.1128/AAC.00590-19 10.1128/AAC.00590-19 31307982 PMC6709465

# Response

**Published:** 2022-09-27

**Authors:** Kevin Wamae

**Affiliations:** KEMRI-Wellcome Trust Research Programme, Kenya

1. Please be accurate and consistent with use of the term “haplotype” and consider revising the manuscript accordingly.

- *We have clarified the use of “haplotype” by settling on the term “microhaplotype” to mean the set of amino acid polymorphisms found on a single DNA amplicon. The term microhaplotype is now a preferred term in similar work.*
***Reference*** *: *
  - *Tessema, S. K., et. al. (2020). Sensitive, Highly Multiplexed Sequencing of Microhaplotypes From the Plasmodium falciparum Heterozygome. The Journal of Infectious Diseases, 1–11.*
  - *LaVerriere, E., et. al. (2022). Design and implementation of multiplexed amplicon sequencing panels to serve genomic epidemiology of infectious disease: A malaria case study. Molecular Ecology Resources, 22(6), 2285–2303.*
  - *Taylor, A. R., Jacob, P. E., Neafsey, D. E., & Buckee, C. O. (2019). Estimating Relatedness Between Malaria Parasites. Genetics, 212(4), 1337–1351.*
  - *WHO: Informal consultation on methodology to distinguish reinfection from recrudescence in high malaria transmission areas. https://www.who.int/publications/i/item/9789240038363*

2. Note that in our publication 1 we used the abbreviation “AmpSeq”, the current authors used “TADS” to mean the same thing. It would be nice to establish a consistent terminology noting the latest WHO recommendation also uses “AmpSeq” i.e.

https://www.who.int/publications/i/item/9789240038363

 If “TADS” is already in the literature I would request (but not demand) they consider changing TADS to Ampseq. Like the use of “haplotype” it is not essential but if a field starts off by using different anacronyms for the same thing it tends to persist which is in nobody’s long-term interest. If “TADS” is not already in use in the literature it would be hard to justify using a new terms when one already exist.

- *Throughout the manuscript, we have switched to using AmpSeq.*

**Methods.**

 2.  I have sat through seeming interminable technical discussions on amplicon sequencing, most of which went over my head. I do know enough to realise it is not as straightforward as most people think so suggest an expert on genotyping also reviews this manuscript. The authors noted that they could detect the minority allele in mixture of 95%:5% and regarded any reads less than 5% of total as noise. We previously found this threshold to be extremely important (i.e. 1) and termed it the Bioinformatics cut-off (BIC), and identified it as a vital piece of information to be included in publications, so it is nice to see it clearly defined here. However, on page 8 it states “our analysis of rare haplotypes was limited to a 5% cut-off based on sequencing controls” so some clarification is needed. Is it 5% of total reads or 5% of controls? If the latter, this could be explained much more clearly.

- *We have clarified that this 5% is based on the proportion of the minor clone in the artificial mixture of the two laboratory clones, that is 95% 3D7 and 5% Dd2.*

4. It looks like the authors extracted their DNA from frozen blood samples. One of the things we previously identified as important was the “blood sampling limit” 1 i.e. the number or density of parasites below which they were unlikely to be detected because the blood volume entering the genotyping assay was so small that parasites of a given genotype were statistically unlikely to be present in the sample. **We made an estimate based on blood extracted from dried blood spots on filter paper**. It looks like the authors need to the same calculations based on the volume of blood they used. This is important for understanding the likely impact of gametocytes, and to interpret observed patterns (i.e., their figure 1) post-treatment; see comment (10) below. **They have total parasitaemia and relative frequencies of ama1 alleles in each patient so they can back-calculate likely number/density of each clone and hence figure out whether it is in danger of falling below the sampling limit**. The calculations tend to be estimates but they need to do this before interpreting their figure 1 i.e. they need to be confident that all the ama1 signals in the patient are being detected and there is **no danger that, at later time points,  one ama1 signal becoming so low (because the clone(s) containing it has fallen to very low density)  that it is no longer detected in the assay despite its continued persistence in the patient**.

 The blood sampling limit is also one plausible explanation for their observation (page 6) that” In five individuals (PIDs 10, 32, 40, 59 and 60), there were sporadic detections of rare *ama1 *haplotypes ” i.e. because parasitaemias of the clone containing that ama1 allele were around the sampling limit so that whether or not they were detected was a matter of chance. The second plausible reason is that it fluctuated around the 5% BIC so sporadically exceed the BIC. Discussion around these points would be good and it also emphasises why it is important to establish the sampling limit before interpreting the results.

- *We have responded to this in comment 10, below.*

5.  I am worried about these statements “The frequency of each haplotype in the population was calculated using the total number of samples that contained the haplotype over the total number of samples genotyped.” This will calculate prevalence *not *frequencies (this has caused immense confusion in the past). The problem can be illustrated by a simple example: assume there are only two samples: sample #1 has alleles A,B and C, and sample #2 has A, B, and D. Then according to their statement the “frequencies” of A, B, C and D will be 100%, 100%, 50% and 50% respectively which is obviously wrong as in this context the allele frequencies should sum to 100%. I assume the statement is written incorrectly and that they counted total number of clones in the two samples i.e. 3+3=6 then calculated the frequencies of A,B, C and D as 2/6. 2/6, 1/6 and 1/6 respectively i.e. ensuring frequencies sum to 100%. They then need, for reasons given below,  to calculate expected heterozygosity (He) to quantify genetic variability ie. the probably that two randomly selected clones have different alleles.

- *We agree with the suggestion. However, we have dropped the statement “The frequency of each haplotype in the population was calculated using the total number of samples that contained the haplotype over the total number of samples genotyped.” This population frequency statistic was not used anywhere in the paper and was erroneously included.*

I also worry about their statement on page 10 when discussing table S2 i.e. “The frequencies were calculated by dividing the number of reads of each haplotype by the total number of reads obtained (116,187,131).” These “frequencies” are likely biased towards alleles in clones in high-parasitaemia patients. So, I think the calculation of frequency suggested above is better, especially because it can be used to calculate H _e_.

  

- *This has been edited to “The frequencies were calculated by dividing the number of reads of each microhaplotype by the **total number of reads obtained per sample**”.*
- *Therefore, we did not use just total reads but total reads per sample. Sequencing libraries from all samples were normalised to equimolar concentrations before pooling and sequencing to avoid having more input DNA from higher parasitaemia samples. Additionally, one of our cut-offs for identifying minor variants required that they were identified in other samples so this reduces bias due to high parasitaemia samples.*

6.  I assume the genotyping at *msp1/msp2/glurp* was the standard WHO-recommended method of gel electrophoresis. As above, the sensitivity (equivalent to the BIC) used in their assay should be stated. Labs typically regard genotyping peaks less than 25% or 30% of the largest peak as noise but there is no consistency in this (and some labs apparently do it “informally”) but this needs to be stated.

- *We have included the following statement in the methods section:*
***“msp1/msp2/glurp genotyping was performed according to the WHO-recommended method of gel electrophoresis*** ***(WHO 2008)*** ***. These data were obtained from the original study, and the following was ensured during the analysis: Each PCR product had to have well-defined and easy to visualise, bands had to be bright and sharp to be of sufficient quality for scoring, PCRs were repeated if bands appeared in the negative control, the interpretation of results did not include products with less than 100 bp and did not account for faint bands or bands that formed smile-shaped patterns on the gel*** ***(Hamaluba et al. 2021)*** ***”***

**Interpretation**

 7. My main concern is that the authors seem to take no account of the possible impact of detecting alleles in gametocytes. We identified  this as a key consideration as illustrated in Figure 2 of our main text and extensively discussed in our Supplementary Material Part 3: “Simulating the potential impact of gametocytes on the accuracy of molecular correction”. 1  The problem is that current drugs clear the asexual form of falciparum but not the gametocytes which continue to circulate and the increased sensitivity of deep sequencing allows detection of alleles in the gametocytes. Suppose a clone initially has 1% gametocytes then after treatment their proportion increases (roughly) 2 fold with every half-life of the asexual forms i.e.  gametocytes increase (approx.) from 1% to 2% to 4% to 8% etc after 1,2,3,4, etc half-lives. The observed clearance half-life will therefore gradually decrease as the drug-insensitive gametocytes make up an increasing proportion of the clone. Whether the gametocytes will be observable in the initial test slide depends on the microscopy and on the clone size. So it’s a two-stage calculation Firstly, the extent to which any putative gametocytes will gradually reduce observed clearance rates post-treatment. Secondly, the likely impact of gametocyte signals will also depend on the blood sampling limit (i.e. whether gametocytes are of sufficient density to de detectable) which I have suggested the authors calculate (cf Fig 2 of Jones *et al*.)

  

- *This has been included as one of our limitations. None of the 30 individuals was gametocyte on day 0 and they remained negative up to day 42. One participant, however, was gametocyte positive on day 3 but negative thereafter so it’s unlikely that gametocytaemia biased our findings.*

8. First paragraph of section “Pfama1 genetic diversity during and after treatment”. The slight decrease in COI is presumably a consequence of clones dropping below the blood sampling limit (see above), or below the 5% BIC,  and being lost. Given that monoclonal infections cannot lose diversity, it would be better to exclude these infections from the calculations to get a better idea of the drop in COI.

  

- *After excluding monoclonal infections, we still see a decrease in COI from before treatment, at 0h the mean COI was 2.44, 0.5h to 72h the mean COI was 2.37 and 2 thereafter. These new values have been included in the text.*

9. I am extremely curious about the assertion made in the caption to figure 2 i.e. “PID32 had the fastest clearing (0.8h) *ama1 *haplotype V9.”. Looking at Figure 1, it appears that PID32 was largely monoclonal except for a single detection of another ama1 allele at time 8hours which may well be spurious. More explanation is needed.

  

- *Regarding the single ama1 microhaplotype detected at 8h, we have shown in extended data table S3 that it was detected above the blood sampling limit. Still, we have no reason to believe that PID32’s infection being clonal would have biased our estimate of determining parasite clearance. *

10. So once background calculation have been made for blood sampling limit and impact of gametocytes there are (as far as I can see) three plausible patterns post treatment.

  

1. Immediate and sustained  difference in clearance rates between clones in the same patient. PID27 therefore looks the most interesting to me as is suggested significant differences in clearance between the two clones immediately post-treatment (Figure 1). Figure 2B also shows that the two clearance rates in this patient are clearly distinguishable on the Y axis unlike the other patients . Note  this could be due to differences in drug sensitivity or, less likely, to differential impact of immunity acting on the two clone.
  - *We did not see a difference in the sensitivity of the drugs; hence we hypothesize that acquired immunity may have had a larger role to play in the difference in clearing times for PID27.*

 

 10. An impact of gametocytes. All clones would retain roughly the same proportions immediately post-treatment, but one eventually rises. The one that increases in proportion could arise as gametocytes become a significant proportion of its biomass (see point 7 above) which are not cleared by the drug . PID08 seems to show this pattern.

*As mentioned above, all participants in the current study were gametocyte negative throughout treatment, thus it’s likely that gametocytaemia biased our findings.*

 Impact of a sampling limit. All clones maintain same proportions until the lower one falls below the sampling limit and disappears, hence the 100% has to be retained by increased proportions on those remaining above the sampling limit. This would be an artefact. Interesting, I can’t see evidence of this of Figure 1 so I’m guessing sampling limit was not a big issue (the requested background calculation should confirm this).

- *We set our arbitrary blood sampling limit to 10 parasites/μl based on the lower limit used in the PMID:* *34252299. In multiclonal infections, some microhaplotypes started to fall below this limit, however, for the seven individuals with microhaplotypes that were detected sporadically, only one (PID60) had a microhaplotype below this limit.*
- *We have included this comment in the discussion, and we recommended future studies to include artificial sequencing-controls with decreasing parasitaemia, e.g. 10,000 down to 1 parasite/μl to determine their limit of detection.*

So these are three paradigms. There may be variation caused by factors such as sequestration,  but given that the authors chose to present and discuss the profiles, I strongly suggest they improve their discussion to include the putative/likely impact of gametocytes and sampling limits.

- *We had mentioned earlier that the sporadic detections of rare microhaplotypes could have originated from changes in parasite densities, sequestered parasites, and lingering nucleic material from dead parasites.*
- *We have also included in our limitations that these rare microhaplotypes could have originated from gametocytes too. However, this was unlikely in this study since all participants were gametocyte negative throughout treatment.*

11. Page 9 they state “Hence, *ama1 *is a highly discriminatory marker with a larger number of variants with a prevalence of <5%, providing a higher resolution to better distinguish new from recrudescent infections in moderate to high transmissions settings where polyclonal infections are common.” This is probably incorrect and definitely incomplete. Msp1, msp2 and glurp are all highly variable, possible more so than ama1: they would need to report H _e_ values for ama1 to substantiate this assertion of greater genetic diversity. The most important point about deep sequencing vs msp1/msp2/glurp is sequencing has much better ability to detect minor clones… they need to cite our previous work (i..e Jones** *et al*.) here as we explicitly discussed the impact of improved sensitivity in detail.

- *This section has been amended to include the high H _e_ we obtained for the ama1 locus we genotyped. Additionally, we have added that amplicon sequencing is more sensitive than msp1/msp2/glurp genotyping because it can better capture minority variants. The section now reads as follows:*
***“hence, the ama1 locus genotyped in this study is a highly discriminatory marker with a larger number of variants with a prevalence of <5% and a high expected heterozygosity value (0.96). Coupled with the high sensitivity of amplicon sequencing to capture minor variants, this assay provides a higher resolution to better distinguish reinfections from recrudescent infections in moderate to high transmissions settings where polyclonal infections are common.”***
